# Rational Design of Supramolecular Receptors for Consistent Binding Affinities under High-Salinity Conditions

**DOI:** 10.1021/acs.joc.5c00068

**Published:** 2025-04-17

**Authors:** Borja Gómez-González, Nuno Basílio, Belén Vaz, M. Rita Paleo, F. Javier Sardina, Moisés Pérez-Lorenzo, Luis García-Río

**Affiliations:** a Department of Physical Chemistry, 16780 Universidade de Santiago de Compostela, Santiago de Compostela 15782, Spain; b Laboratório Associado para a Química Verde (LAQV), Rede de Química e Tecnologia (REQUIMTE), Departamento de Química, Faculdade de Ciências e Tecnologia, Universidade NOVA de Lisboa, Caparica 2829-516, Portugal; c CINBIO, 16784 Universidade de Vigo, Vigo 36310, Spain; d Galicia Sur Health Research Institute, Vigo 36310, Spain; e Center for Research in Biological Chemistry and Molecular Materials (CIQUS), 16780 Universidade de Santiago de Compostela, Santiago de Compostela 15782, Spain; f Department of Organic Chemistry, 16780 Universidade de Santiago de Compostela, Santiago de Compostela 15782, Spain; g Centro de Investigación Mestrelab (CIM), Av. Barcelona 7, Santiago de Compostela 15706, Spain

## Abstract

The development of water-soluble multicharged macrocycles has opened promising pathways in biomedical applications, enabling selective molecular recognition for therapeutic and diagnostic uses. Yet, traditional polyanionic and polycationic receptors often face performance limitations under realistic operating conditions. A major drawback is the natural tendency of these polycharged hosts to experience increasing screening effects as concentration rises due to self-ion pairing phenomena, which can reduce binding efficiency by several orders of magnitude. These issues are further intensified when polyionic receptors are used in high-salinity environments, typically used to replicate physiological settings, where the abundance of ions introduces additional screening effects that diminish the supramolecular affinity for a wide range of guests. This study presents a new approach that leverages zwitterionic synthetic receptors with rationally engineered architectures to overcome these challenges. By incorporation of specific structural features, self-ion pairing is eliminated, effectively making host concentration no longer a controlling factor in the thermodynamics of the complexation process. Additionally, these dual-charged hosts achieve self-contained stabilization, naturally shielding recognition sites from external ion interference under high-salinity conditions. Furthermore, the ability of these supramolecular hosts to encapsulate zwitterionic guests, a challenging task due to the strong solvation of these molecules in aqueous solution, adds significant value to the functional versatility of these macrocycles. Altogether, these findings represent a significant advancement in the design of stable and adaptable receptor systems for complex environments.

## Graphical abstract

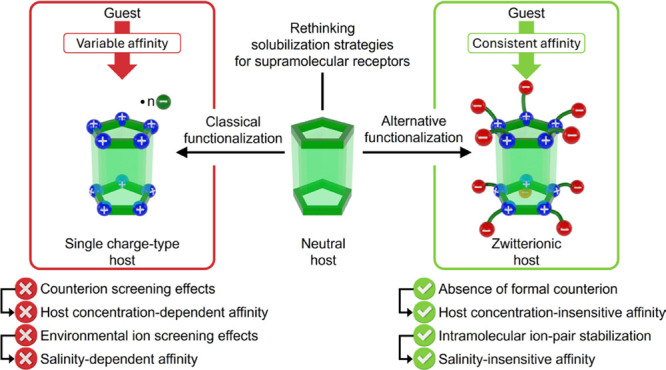

## Introduction

Over the years, the design of macrocyclic supramolecular hosts has attracted considerable interest because of their extensive potential in numerous biomedical applications.^1–4^ These synthetic receptors find use in contexts including drug delivery,^5–8^ bioimaging,^9–11^ biosensing,^12–14^ theranostics,^15–17^ disease inhibition,^18,19^ antimicrobial treatments,^20–22^ drug sequestration,^23–25^ cell biomimicry,^26,27^ or tissue engineering.^28,29^ Given these diverse bioapplications, it is essential that these artificial hosts are functional in aqueous environments.^30,31^ This requirement becomes especially crucial for aromatic receptors, such as those belonging to the large cyclophane family.^32^ Unlike other well-known macrocycles, such as cyclodextrins, which naturally possess water-soluble functional groups, cyclophanes generally exhibit intrinsically low water solubility. This hydrophobic nature restricts their utility in biological systems, where water solubility is a key prerequisite. Consequently, it is often necessary to make use of functionalization strategies to enhance their water compatibility, enabling their practical application in realistic biological contexts.^33^

Within the cyclophane family, pillararenes stand out due to their remarkable synthetic versatility, which allows for the relatively straightforward incorporation of functional groups that provide the desired water solubility.^34^ Several synthetic approaches can be applied, including mono-,^35^ di-,^36,37^ and tetra-functionalization,^38,39^ together with rim differentiation,^40,41^ lateral functionalization,^42,43^ and phenylene ortho-substitution.^44,45^ Still, one of the most widely adopted methods is the per-functionalization strategy, which typically involves decorating both the upper and lower rims of the hydrophobic cavity with multiple (identical) charged groups.^34^ This approach is especially advantageous because the high symmetry of the final products helps to avoid complex mixtures and the associated separation challenges. Moreover, the proximity of these charged groups to the hydrophobic cavity may create a synergistic effect, where hydrophobic interactions within the cavity and electrostatic interactions at the portals work together.^46^ This combination facilitates highly efficient molecular recognition between pillararenes and guests containing polar and nonpolar domains, thus creating a versatile system for aqueous environments.

At this point, it is important to highlight that incorporating multiple charged groups at the portals of these macrocycles may come with several drawbacks that can compromise their complexation ability and consequently hinder the binding process with potential guests. One significant issue is the introduction of counterions, which inevitably accompanies the incorporation of water-solubilizing ionic moieties. This can lead to notable screening effects, as even at low host concentrations, substantial self-ion pairing can occur.^47^ This interaction creates a highly complex scenario in which polycharged receptors can simultaneously adopt different electrostatic configurations, resulting in varying supramolecular affinities for a given guest molecule. Specifically, although highly charged configurations are predominant at low macrocycle concentrations, less charged versions dominate at higher concentrations. These distinct degrees of neutralization of the receptor’s portal charges profoundly impact binding affinity, especially when electrostatic forces play a key role in the host–guest interaction. In this regard, it is important to note that although this effect is often overlooked in water due to its high dielectric constant, reductions in complexation affinities by up to 6 orders of magnitude have been reported for anionic guests and deca-trimethylammonium pillar[5]­arene bromide salts.^47^ Notably, this effect has also been observed in scenarios involving cationic guests in the presence of sulfonatocalix[4]­arenes featuring counterions of different nature, where increases in host concentration lead to a one-order-of-magnitude decrease in supramolecular affinity.^48,49^ Another major concern is that synthetic receptors designed for biomedical applications are typically studied in buffered solutions or in the presence of added salts to simulate the physiological pH and ionic strength. Similarly, these protocols often undermine the binding process, as the large excess of ions added to the solution can lead to significant screening effects, ultimately resulting in a reduced efficiency of the supramolecular binding events.^50^ This phenomenon has been observed in sodium sulfonatecalixarenes, where the introduction of excess Na^+^ ions results in a two-order-of-magnitude decrease in the supramolecular binding constant.^49,51^ Ultimately, the extent of this effect depends on the specific interactions between the host and its counterions, the competitive equilibria with the guest, and the overall experimental conditions that influence these interactions.^52^ Therefore, each system should be analyzed individually to accurately evaluate its thermodynamic properties.

In response to these challenges, there is a pressing need for novel synthetic strategies to create artificial receptors that retain their operational features, regardless of working concentrations or their use in high-salinity environments and biological fluids.^33^ For this reason, we propose the rational design of a multicharged macrocycle whose architecture is resilient to the disruptive effects of self-ion-pairing or external ion-pairing phenomena, ensuring that its binding capacity remains consistent despite variations in host content or their use under conditions that mimic physiological settings. To achieve this, a pillar[5]­arene receptor is constructed that features no formal counterions, thereby avoiding self-ion pairing phenomena. Additionally, it incorporates zwitterionic functionalities designed to provide the cyclophane with self-contained stabilization, avoiding the need for interaction with competing ions. Ultimately, this design enables the system to maintain robust supramolecular affinities. Successfully achieving this stability in challenging settings, such as biofluids, would address long-standing limitations in host–guest chemistry under physiologically relevant conditions. This advancement could have far-reaching implications, not only enhancing the applicability of macrocyclic hosts in clinical and therapeutic settings but also opening new avenues for their use in other high-salinity environments, where molecular interactions can be inhibited. These include industrial processes such as wastewater treatment,^53^ oil recovery,^54^ or electrochemical energy storage,^55^ as well as the fabrication of smart organic materials.^56^

## Results and Discussion

The synthesis of the perfunctionalized zwitterionic pillar[5]­arene (**ZP5A**) is accomplished following the strategy partially outlined in Scheme 1 (see the Supporting Information for further details). At this point, it is crucial to emphasize that several key factors must be carefully considered in advance to ensure a reliable performance of this receptor. One critical detail is that the ionic groups responsible for the macrocycle’s solubility must either carry permanent charges or possess sufficiently extreme p*K*_a_ values to remain ionized across the different pH levels encountered in physiological conditions.^57^ In this case, the quaternary ammonium and sulfonate groups fulfill these requirements. Another important feature is that the supramolecular host must lack formal counterions to avoid self-ion-pairing effects, which often compromise the binding efficiency of multicharged systems in aqueous environments. This can be achieved by incorporating the zwitterionic functionalities through reactions in which the precursors are neither ionic in nature nor possess groups that, upon heterolytic bond cleavage, would yield charged leaving groups.^58^ In this case, both the Eschweiler–Clarke methylation of primary amines using an excess of formic acid and formaldehyde and the subsequent ring-opening reaction of 1,3-propane sultone effectively meet these requirements. The initial reductive amination prevents the formation of quaternary ammonium salts, while the *N*-alkylation proceeds, avoiding the formation of counterions during the process. A further crucial aspect of the design is the nature of the linker that connects the positive and negative charges within the zwitterionic chains, functionalizing the portals of the macrocycle. The length of this spacer must provide enough structural flexibility to enable the chain to loop back on itself or interact with neighboring chains, thus promoting the formation of intramolecular ion pairs. This partial neutralization of the charges avoids the need for association with external ions that would otherwise be responsible for stabilizing the structure, particularly in high-salinity environments. Here, the length of the zwitterionic chain suggests that such folding is indeed feasible, allowing for a partial offset of the charges among the multiple functional groups of the macrocycle. In line with this requirement, it is also essential that the ionic groups within the zwitterionic functionalities exhibit similar dimensions. According to the law of matching water affinities (LMWA), ions of similar size exhibit comparable hydration energies, which favor the formation of small–small or large–large ion pairs.^59^ In contrast, ions of different sizes generally display weaker electrostatic attractions. Given the similar ionic radii of the functional groups incorporated into **ZP5A** (quaternary ammonium and sulfonate ions), intramolecular electrostatic interactions are expected to be favored.

**Scheme 1:**
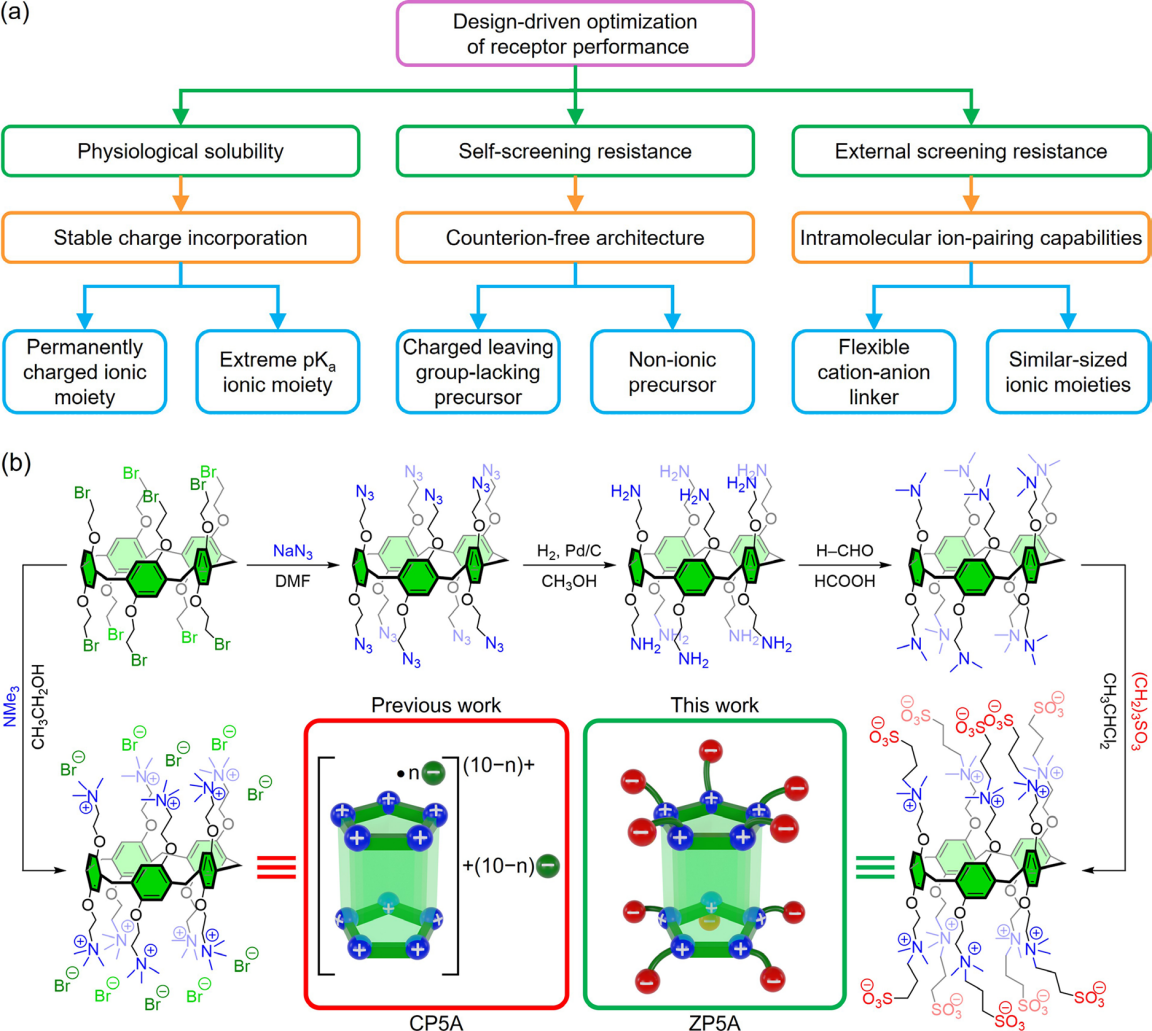
(a) Flowchart Depicting the Key Structural Elements in the Design of Supramolecular Receptors with Efficiencies that Remain Unaffected by Varying Host Concentrations or the Presence of High-Salinity Environments, and (b) Synthetic Strategy for the Preparation of a Water-Soluble Pillararene Endowed with Zwitterionic Functionalities and Comparative with Previous Work

After the zwitterionic pillar[5]­arene was synthesized, a preliminary assessment was performed to ensure that incorporating charged functional groups into the aromatic structure leads to no colloidal aggregation. To this end, diffusion-order spectroscopy (DOSY) is used to evaluate any potential self-assembly based on the analysis of the diffusion coefficients of **ZP5A** (Figures S22–S24). As shown in Table S1, this parameter remains fairly constant across the working concentration range, indicating the absence of aggregation phenomena. Following this validation, it is essential to assess the impact of incorporating zwitterionic functionalities on the selectivity and operational capabilities of this supramolecular receptor. In this regard, the dual nature of **ZP5A**, featuring both nonpolar and polar-charged domains, suggests that this macrocycle possesses a robust capacity to interact with a diverse range of ionic organic molecules. To assess the specificity of the host, a selection of organic guests with varying charge distributions is employed, allowing for a comprehensive exploration of how electrostatic and hydrophobic interactions contribute to the binding affinity. In pursuit of this goal, NMR spectroscopy is applied due to its great potential to provide detailed information on molecular interactions, binding sites, and conformational dynamics in host–guest systems.^60^

The first guest selected is *p*-toluenesulfonate (**TS** ^**–**^). As shown in Figure 1, when 1 equiv of **ZP5A** is added to a solution of **TS** ^–^, the methyl group signal of the guest is significantly upfield-shifted (Δδ = −1.77 ppm) due to the ring current effect of the aromatic inner space. This indicates the formation of a **ZP5A:TS** ^**–**^ complex, in which the aromatic ring of **TS** ^**–**^ is deeply embedded within the π-electron-rich internal cavity of the pillararene. This binding event likely arises from π–π and CH−π interactions between the macrocycle and the guest molecule. The upfield shift is less pronounced for the protons located ortho to the sulfonate group of **TS** ^**–**^ (Δδ = −0.18 ppm), suggesting that they are not as deeply inserted in the receptor’s cavity. Thus, it is reasonable to assume that the −SO_3_^–^ group is positioned in close proximity to the quaternary ammonium groups at the portals of the macrocycle, further stabilizing the inclusion complex through attractive electrostatic interactions. It is important to note that the behavior of the zwitterionic pillar[5]­arene in the presence of organic anions mirrors that previously reported for its cationic counterpart, **CP5A** (Scheme 1).^47^ This similarity in behavior is logically expected, as in the current configuration, the positive charges in **ZP5A** are situated at the portals of the aromatic cavity, analogous to their positioning in **CP5A**. This specific orientation promotes synergies where electrostatic interactions cooperate with hydrophobic effects to enable more efficient molecular recognition.

**Figure 1:**
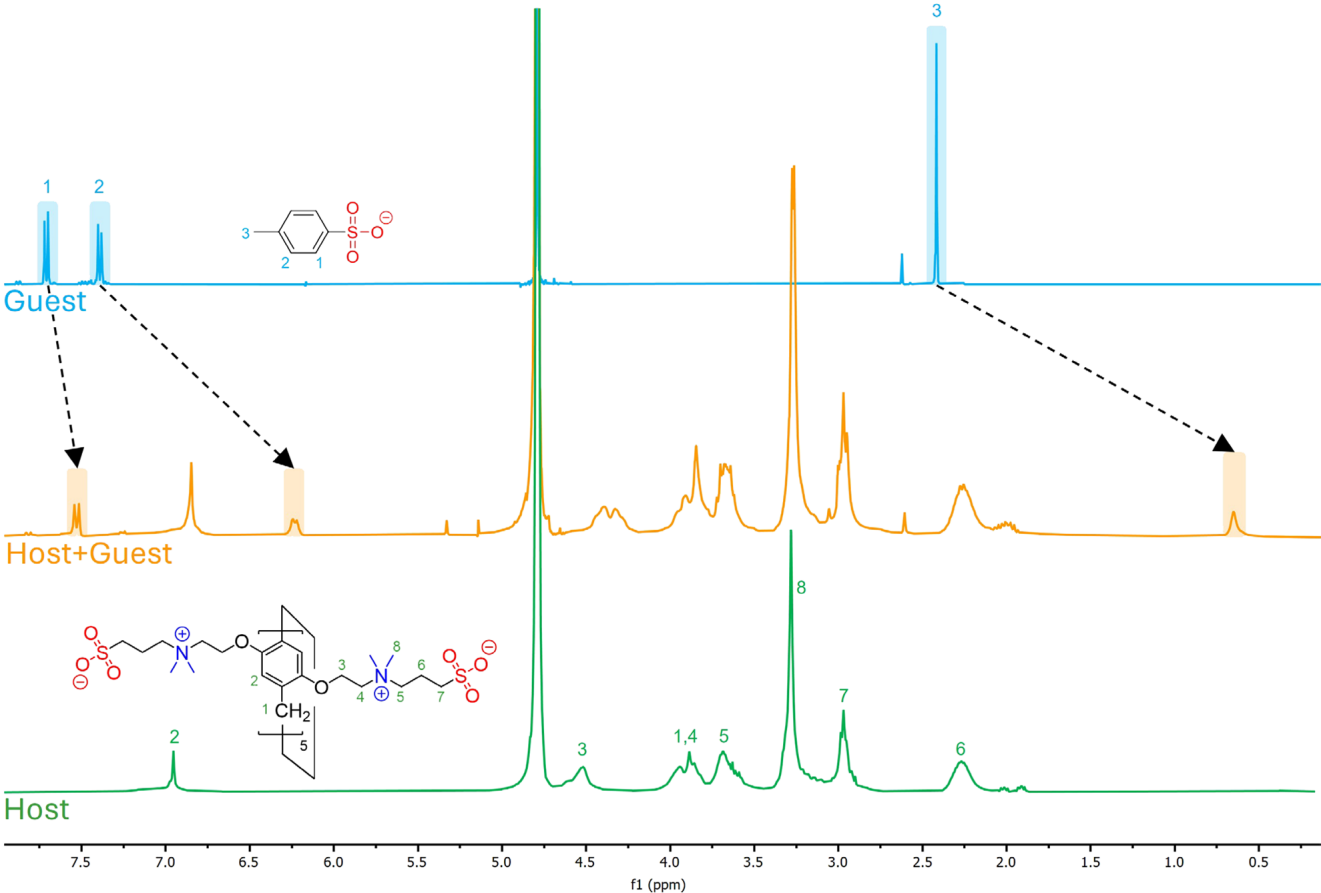
Set of ^1^H NMR spectra in D_2_O at 25.0 °C of: (a) *p*-toluenesulfonate ([**TS** ^**–**^] = 2.0 mM), (b) mixture of zwitterionic pillararene ([**ZP5A**] = 2.0 mM) and *p*-toluenesulfonate ([**TS** ^**–**^] = 2.0 mM), and (c) zwitterionic pillararene ([**ZP5A**] = 2.0 mM).

In order to complement the results obtained via ^1^H NMR, isothermal titration calorimetry (ITC) is also employed (see the Supporting Information for further details). This technique provides direct measurements of the heat changes during the binding process, offering essential insights into the binding mode, as well as key thermodynamic parameters such as binding affinity, enthalpy, and entropy in a single experiment.^61^ These data allow for a deeper understanding of how the host and guest interact within the inclusion complex and offer valuable information regarding the stability of the host–guest arrangement. In this case, the calorimetric titration of **ZP5A** with **TS** ^**–**^ fits accurately to a 1:1 “one set of binding sites” model (Figure S25). This approach enables the determination of the thermodynamic parameters governing the binding event, revealing an affinity constant of *K*_**ZP5A**:**TS**_^*–*^ = (6.8 ± 0.1) × 10^3^ M^–1^. As initially suggested by the NMR results, this significant value can be attributed to the strong complementarity between the host and guest, primarily driven by various noncovalent interplays, including electrostatic forces, π–π stacking, and CH−π interactions. Consequently, in light of the numerous favorable interactions between **ZP5A** and **TS** ^**–**^, it is reasonable to foresee that the complexation process is primarily enthalpy-driven. This is evidenced by the dominant contribution of enthalpy (Δ*H* = −15.8 ± 0.1 kJmol^–1^) to the free energy of the process, which outweighs the favorable entropic change (*T*Δ*S* = +5.97 ± 0.50 kJmol^–1^). In this case, the observed entropic contribution is characteristic of the classical hydrophobic effect, resulting from the release of water molecules into the bulk solvent as hydrophobic surfaces come into contact, reducing the water-solvated surface area.

Once the binding constant between **ZP5A** and **TS** ^**–**^ is determined, it becomes crucial to assess whether the presence of excess salts negatively influences the macrocycle’s affinity for the guest molecule. As previously noted, introducing ions into the solution to mimic physiological conditions often adversely impacts the formation of host–guest complexes, particularly when electrostatic interactions are key. To explore this, calorimetric titrations are performed by introducing a substantial excess of NaBr (Figure S26) and NaBF_4_ (Figure S27). Table 1 illustrates that the affinity constants obtained from these experiments remain fairly unchanged, confirming that the complexation process is resilient to the high salinity of the environment. The consistent binding observed in the presence of salts can be rationalized by the formation of intramolecular ion pairs within the zwitterionic receptor. The considerable structural flexibility of the linker connecting the positive and negative charges of the macrocycle facilitates both intrachain and interchain interactions, resulting in effective self-contained stabilization of the multicharged pillararene in aqueous solution. This conformational flexibility facilitates interactions between similarly sized quaternary ammonium and sulfonate ions. In the presence of NaBr, and as predicted by LMWA, these interplays are favored over those with the smaller bromide ion. This dynamic prevents Br^–^ from associating with the macrocycle’s portals, effectively mitigating the screening effects that typically hinder the formation of host–guest complexes, especially in situations where electrostatic interactions are critical. These internal interactions are also predominant when an excess of BF_4_^–^ is present. Initially, it could be expected that the larger size of BF_4_^–^ compared to that of Br^–^ would strengthen its interaction with the positive charges of the macrocycle. However, the high local concentration of sulfonate moieties, which are physically bound around the receptor’s portals, appears to be sufficient to counterbalance the influence of excess interfering anions. These findings starkly contrast with those determined calorimetrically for the analogous pillar[5]­arene, which is exclusively cationic in nature (Figures S28–S30). As shown in Table 1, for the same concentration of host, the average affinity constant between **CP5A** and **TS** ^**–**^ significantly diminishes in the presence of excess Br^–^, decreasing by a factor of 3. This reduction is even more pronounced in the presence of an excess of BF_4_^–^, where the affinity constant is reduced by a factor of 8. This pronounced screening effect observed with BF_4_^–^ is expected, as the LMWA indicates that this anion exhibits a greater affinity for the quaternary ammonium groups of **CP5A** compared to that of Br^–^. Based on the results presented in Table 1, the advantages of employing zwitterionic pillararenes for encapsulating organic anions become evident, as the self-stabilization of the macrocyclic structure effectively mitigates the common screening effects encountered in high-salinity solutions. This ability to counteract such effects is further confirmed through titration experiments conducted at 137 mM NaCl, where it is shown that the affinity constant of ZP5A remains stable even at biologically relevant salinity levels (Figure S31). It is also important to note that this consistency in supramolecular affinity appears to come with certain trade-offs. Although the binding constants remain stable and relatively high in value, they are lower than those observed for the exclusively cationic pillar[5]­arene. This reduction can be attributed to a degree of charge attenuation at the portals due to the formation of intramolecular ion pairs. Additionally, the spatial arrangement of the zwitterionic chains surrounding the macrocycle’s portals may introduce some steric hindrance, impeding the inclusion of organic anions compared to the more exposed cavity of **CP5A**. In any case, the operational stability of **ZP5A**, combined with the magnitude of the binding constants, demonstrates that the inclusion complexes are still strongly favored, positioning the incorporation of zwitterionic moieties as a promising alternative to traditional strategies for the per-functionalization of supramolecular macrocycles.

**Table 1:**
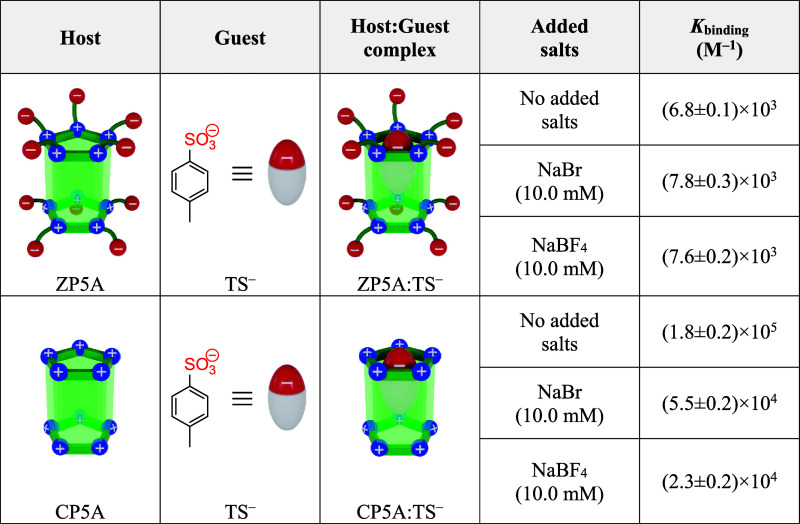
Supramolecular Affinity Constants Determined by ITC for the Complexation of *p*-Toluenesulfonate with the Zwitterionic and Cationic Pillar[5]­arene (Averaged), Evaluated Both in the Absence and Presence (10.0 mM) of Inorganic Salts

At this stage, to provide further evidence supporting the previous observations, increasing concentrations of **ZP5A** are introduced into a solution containing a fixed amount of NaBr. The extent of ion pairing between **ZP5A** and Br^–^ is assessed by measuring the concentration of the remaining free bromide ions in the solution. This is accomplished using a bromide-selective ion electrode, which enables accurate detection and quantification of the unbound Br^–^.^62^ As illustrated in Figure 2, the concentration of free Br^–^ remains unchanged, even when the concentration of quaternary ammonium groups, potentially capable of interacting with Br^–^, is over 30 times higher than that of NaBr in the solution. The lack of ion-pairing between the bromide anions and the positive charges of the macrocycle strongly indicates that the zwitterionic host is indeed self-stabilized through intramolecular electrostatic interactions. This finding sharply contrasts with the behavior observed with the purely cationic pillar[5]­arene. Here, a substantial portion of bromide ions, inevitably introduced as counterions during the incorporation of the quaternary ammonium groups, remains bound to the macrocycle. This association of Br^–^ with the macrocycle partially neutralizes the positive charges at the portals, ultimately diminishing the overall supramolecular affinity of the macrocycle.^47^

**Figure 2:**
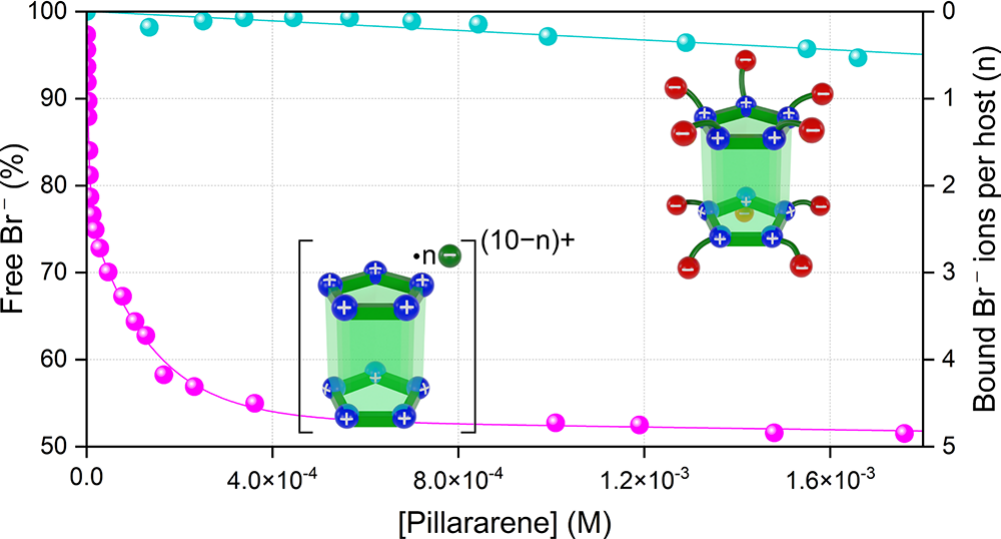
(Cyan) Percentage of free bromide ion as a function of **ZP5A** concentration ([NaBr]_0_ = 6.5 × 10^–4^ M), and (pink) percentage of free bromide ion as a function of **CP5A** concentration (with Br^–^ present as the counterion).

After demonstrating the exceptional ability of the zwitterionic pillar[5]­arene to form complexes with organic anions, even under high-salinity conditions, the next step is to assess whether **ZP5A** can also encapsulate organic cations. To test this, benzyltrimethylammonium (**BTA** ^**+**^) is chosen as a potential guest, and its interaction with the macrocycle is analyzed using ^1^H NMR, similar to the previous experiments with anions. As shown in Figure S32, no shifts are observed in the signals of the cation, ruling out the formation of an inclusion complex. This behavior can be attributed to the host’s architecture, where the cationic groups at the portals are located near the hydrophobic cavity. The inclusion of the aromatic ring of **BTA** ^**+**^ would cause the trimethylammonium functionality to come into close contact with the quaternary ammonium groups at the portals, leading to unfavorable electrostatic repulsion. These findings emphasize the importance of synergistic interactions between electrostatic forces and π-effects in defining the macrocycle’s selectivity.

At this point, considering the results obtained with both organic anions and cations, it becomes essential to examine **ZP5A**’s ability to interact with zwitterionic guests. This could provide deeper insights into the complementary interactions between the dual-charged nature of both the host and guest molecules. This analysis is particularly important because zwitterionic species in aqueous environments typically exhibit a low tendency to form inclusion complexes due to their high hydration levels and stabilization. Therefore, designing supramolecular receptors with a strong affinity for zwitterionic entities remains a key challenge and an area of significant interest in molecular recognition.^63^ To evaluate the capacity of **ZP5A** to accommodate zwitterions, three compounds are selected: 3-(*N*,*N*-dimethyloctylammonium)­propanesulfonate (**Z1**), octyl-(2-(trimethylammonium)­ethyl)­phosphate (**Z2**), and hexyl-(2-(butylammonium)­ethyl)­phosphate (**Z3**) (Figure 3). These species share common structural features. All three possess long hydrocarbon chains, which should favor the inclusion of these zwitterions in the hydrophobic cavity of **ZP5A**. Additionally, they exhibit a similar distance between their cationic and anionic charge centers, facilitating a more effective comparison. One of the primary differences among the guest molecules lies in the geometric arrangement of their charges relative to their nonpolar domain. For **Z1**, the cationic group is adjacent to the hydrocarbon chain, while the anionic group is located at the terminal position. In contrast, for **Z2** and **Z3**, the anionic group is positioned adjacent to the nonpolar region of the guest molecule, while the cationic group is located farther from the hydrophobic domain, being at the terminal end in **Z2** and at an internal position along the chain in **Z3**. In addition, a critical difference between **Z2** and **Z3** lies in the ability of **Z3** to establish hydrogen bonds through its −NH_2_^+^– functionality. This unique feature allows for the investigation of synergies arising from the simultaneous formation of electrostatic interactions and hydrogen bonding between the guest and the host, which may result in more efficient molecular recognition.

**Figure 3:**
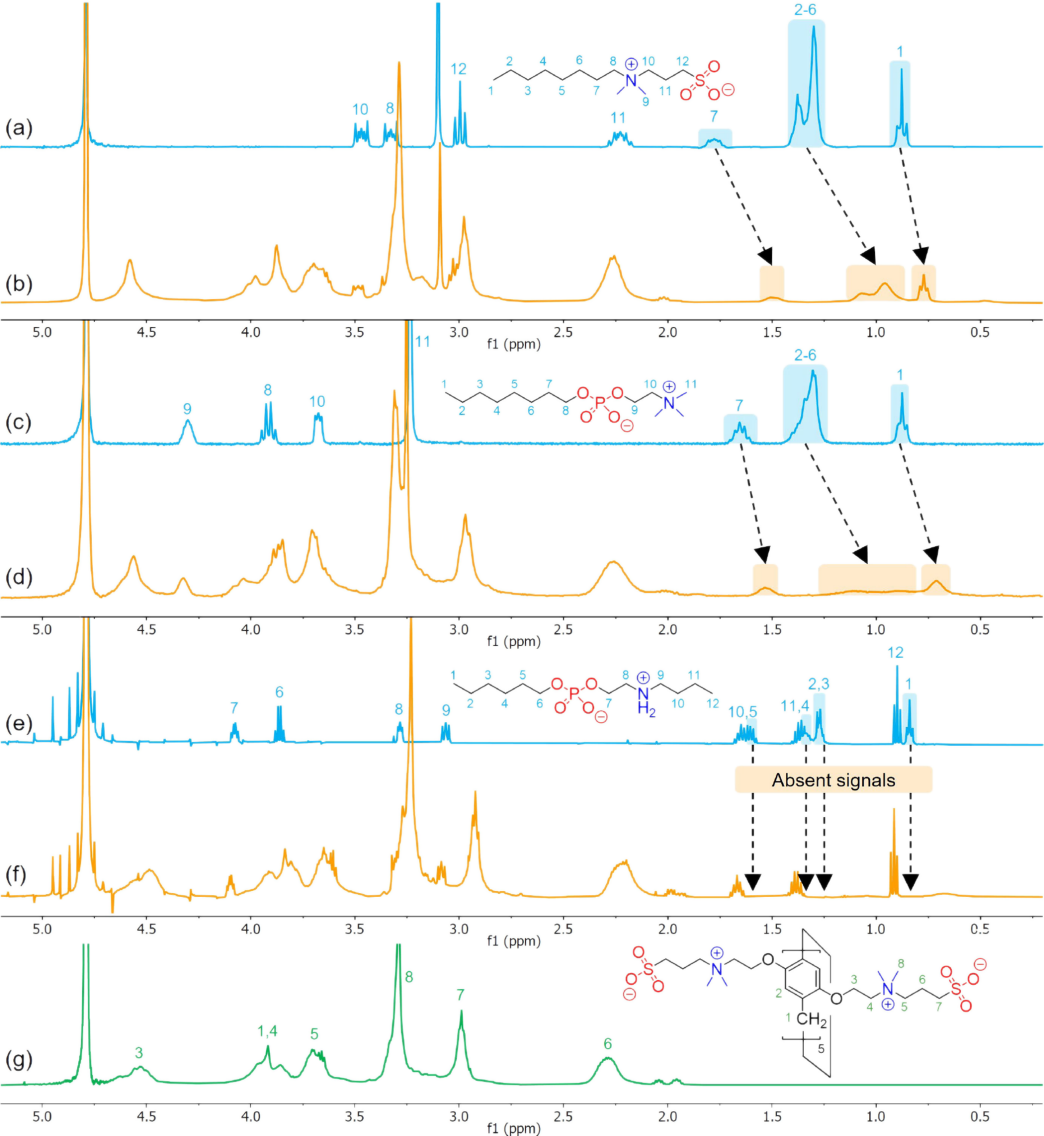
Set of ^1^H NMR spectra in D_2_O at 25.0 °C of (a) 3-(*N*,*N*-dimethyloctylammonium) propanesulfonate ([**Z1**] = 7.0 mM), (b) mixture of zwitterionic pillararene ([**ZP5A**] = 7.0 mM) and 3-(*N*,*N*-dimethyloctylammonium)­propanesulfonate ([**Z1**] = 7.0 mM), (c) octyl-(2-(trimethylammonium)­ethyl)­phosphate ([**Z2**] = 4.0 mM), (d) mixture of zwitterionic pillararene ([**ZP5A**] = 4.0 mM) and octyl-(2-(trimethylammonium)­ethyl)­phosphate ([**Z2**] = 4.0 mM), (e) hexyl-(2-(butylammonium)­ethyl)­phosphate ([**Z3**] = 4.0 mM), (f) mixture of zwitterionic pillararene ([**ZP5A**] = 4.0 mM) and hexyl-(2-(butylammonium)­ethyl)­phosphate ([**Z3**] = 4.0 mM), and (g) zwitterionic pillararene ([**ZP5A**] = 2.0 mM).

As an initial step, and following the same approach applied to the previous guests, the interplay between **ZP5A** and **Z1** is analyzed via ^1^H NMR spectroscopy. As shown in Figure 3a,b, the most prominent chemical shift changes upon host–guest interactions occur in the hydrocarbon chain of **Z1** (H1–H7). The upfield shift of these protons, consistent with aromatic ring current effects, indicates the encapsulation of the guest’s nonpolar domain within the hydrophobic cavity of the host. Additionally, a notable broadening of these proton signals is detected, which is expected given the dramatic impact of supramolecular inclusion on the relaxation times of the guest’s protons.^64^ Furthermore, the remaining **Z1** signals remain unaffected, suggesting that protons outside the hydrophobic tail are located far from the binding site. A similar trend is observed when analyzing the chemical shifts of **Z2** and **Z3** in the presence of **ZP5A**. In this case, significant broadening of the hydrophobic tail protons (H1–H7) is detected for **Z2** upon binding (Figure 3c,d). This effect is even more pronounced for **Z3**, where the signals corresponding to the hydrocarbon chain protons (H1–H5) completely disappear upon complex formation (Figure 3e,f). These findings collectively confirm the close contact between **ZP5A** and the evaluated organic zwitterions, underscoring the critical role of the guests’ nonpolar domains as essential structural motifs for the formation of stable host–guest complexes.

Once this qualitative information is obtained, it becomes essential to quantify the stability of the formed complexes. To achieve this, ITC experiments are performed. The calorimetric titrations of **ZP5A** with the zwitterionic guests **Z1** (Figure S33), **Z2** (Figure S34), and **Z3** (Figure S35) all fit accurately to a 1:1 “one set of binding sites” model, thereby providing the thermodynamic parameters that govern the complexation events (Table 2). Several conclusions can be drawn from analyzing the binding constants obtained through ITC. First, the magnitude of these parameters is noteworthy, consistently exhibiting values on the order of 10^3^ M^–1^. This is particularly significant considering the substantial desolvation penalty that zwitterions incur, which must be compensated by the noncovalent interactions established within the supramolecular complex. Moreover, it is crucial to highlight the differences in how strongly the various guests bind to the pillararene within this range of affinities. Notably, the equilibrium constant for the formation of the **ZP5A:Z2** complex is nearly twice that of **ZP5A:Z1**. Understanding this increase requires considering the insertion mode of the zwitterions, as previously elucidated by ^1^H NMR experiments. Both **Z1** and **Z2** enter the hydrophobic cavity through their hydrocarbon chains. However, only **Z2** enables a supramolecular arrangement where the guest’s charges complement those at the portals of the macrocycle, enhancing the binding interaction. The distinct enthalpic changes observed in these two processes further support this interpretation. The inclusion of **Z2** in **ZP5A** is highly exothermic, which can be attributed to the dual ionic interactions between the charges of **Z2** and the pillararene portals, combined with CH−π interactions between the hydrophobic chain of the guest and the receptor’s cavity. In contrast, the reduced enthalpic change observed for the formation of the **ZP5A:Z1** complex reflects a less favorable charge arrangement, limiting attractive forces to mostly CH−π interactions. The entropic term of the binding free energy also seems to corroborate the hypotheses proposed regarding the binding modes. While the formation of **ZP5A:Z1** is associated with a favorable entropic change derived from the classical hydrophobic effect, the formation of **ZP5A:Z2** involves a negative entropic variation. This unfavorable contribution can be attributed to the significant immobilization of **Z2** within the macrocyclic cavity, where it is strongly anchored through two electrostatic points of interaction, effectively restricting the guest’s degrees of freedom upon binding.

**Table 2:**
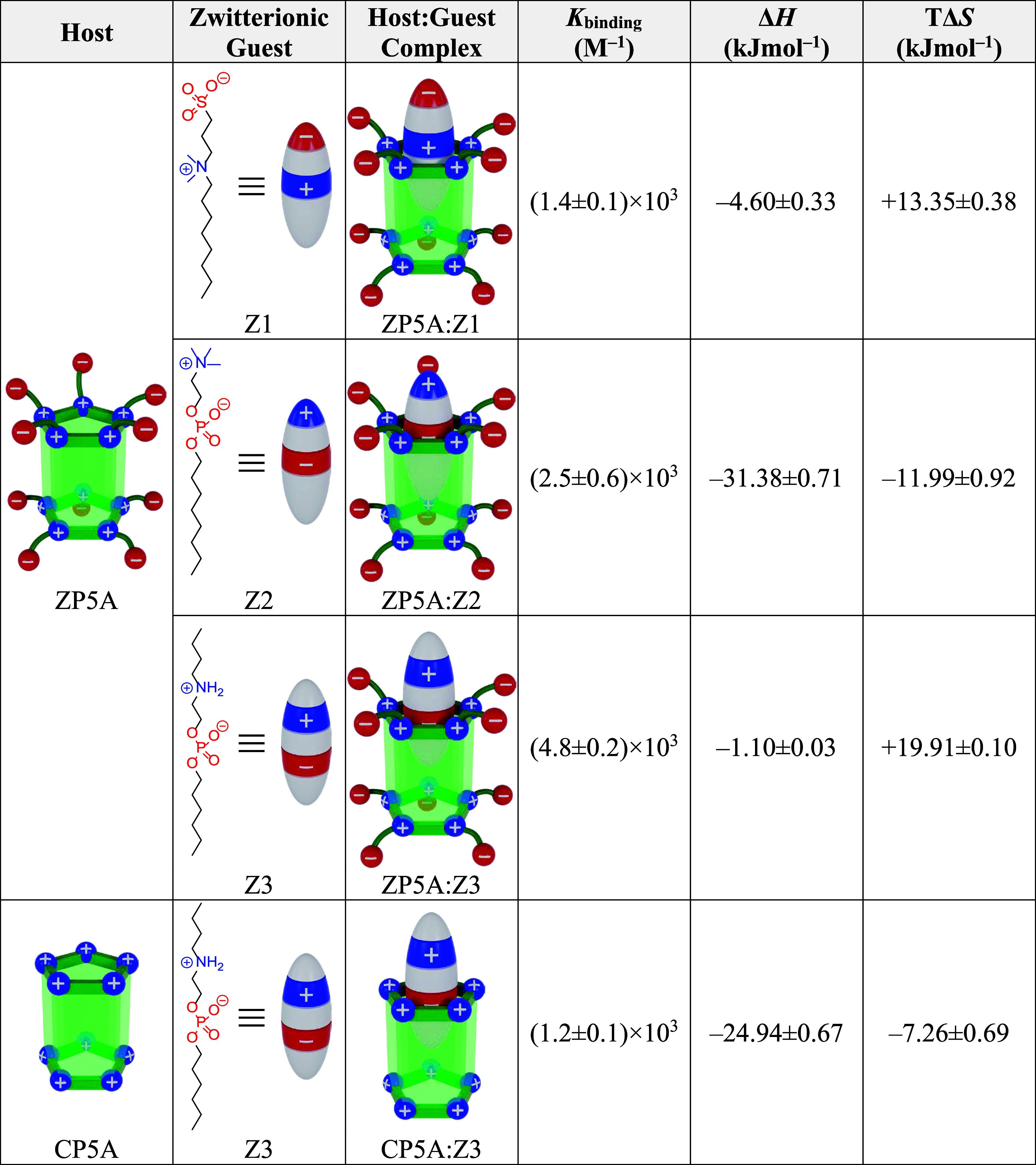
Molecular Structures of the Zwitterionic Guests Used in This Study alongside a Tentative Schematic Representation of Their Interactions with **ZP5A** as Inferred from the NMR Data, and Thermodynamic Parameters for The Complexation of These Guests with **ZP5A** and **CP5A** Determined by ITC Measurements

At this point, it is necessary to analyze in detail the binding process for **Z3**, given the additional potential of this zwitterion to establish hydrogen bonds, which may confer greater stability to the resulting supramolecular complex. In this case, the formation of the **ZP5A:Z3** complex exhibits a binding constant nearly double that of the **ZP5A:Z2** complex. Since the anionic group is common to both **Z2** and **Z3**, the more favorable formation of the **ZP5A:Z3** complex can only be attributed to the stronger interaction between the protonated secondary amine of **Z3** and the sulfonate groups of **ZP5A**. In this regard, this interaction involves both electrostatic forces and hydrogen bonding (i.e., a salt bridge) compared to the purely ionic interaction between the quaternary amine of **Z2** and the anionic groups of the pillararene. In this context, as shown in Table 2, it is important to highlight that the greater number of noncovalent interactions **Z3** can establish (compared to **Z2**) does not lead to an increase in the enthalpic change. On the contrary, the enthalpic variation is significantly reduced, suggesting the occurrence of a prior endothermic process. Given the high structural flexibility of **Z3**, it is plausible to hypothesize the preexistence of a robust intramolecular hydrogen bond in this guest. Thus, the formation of the **ZP5A:Z3** complex should be understood as a host-induced reorganization of **Z3**, involving the breaking of the intramolecular H-bond in the guest (Δ*H* > 0) prior to the binding event (Δ*H* < 0). Ultimately, this energy compensation would render the binding process strongly reliant on the entropic contribution, which, as shown in Table 2, is clearly favorable for the formation of the supramolecular complex. In this scenario, the high affinity of **Z3** for **ZP5A** can be attributed to the increase in degrees of freedom arising from the disruption of hydrogen-bonding constraints upon binding. This effect may offset the entropy loss that is typically associated with complex formation, leading to a net entropic increase.

To further support the above assumption, the binding process between **CP5A** and **Z3** is analyzed calorimetrically (Figure S36). As shown in Table 2, the absence of anionic functional groups at the portals of this pillararene has a noticeable effect on the binding event. This is because a single type of electrostatic interaction can occur between the host and the guest, thereby reducing the affinity of **Z3** for **CP5A** compared to that observed for **ZP5A**. In any case, it is important to note that this comparison is made in the millimolar concentration range, where the electrostatic charge of CP5A is partially screened, leading to a lower supramolecular binding constant.^47^ Alternatively, if the comparison between ZP5A and CP5A were conducted in the micromolar concentration range, CP5A would likely exhibit a higher binding constant relative to ZP5A, as the cationic pillararene would retain a higher effective charge under these conditions. This increased charge could ultimately compensate for the fact that Z3 interacts with CP5A solely through electrostatic interactions. From an enthalpic perspective, the complexation with the cationic pillararene is highly exothermic. This finding rules out the occurrence of energy compensation phenomena, given that no intermolecular interaction exists to disrupt the intramolecular hydrogen bond of **Z3**, which remains intact. The preservation of H-bonding logically impacts the entropic term of the free energy as the structural constraints of the guest are maintained. Consequently, a negative entropy variation is observed in this case, although it is somewhat less pronounced than in the **ZP5A:Z2** complex. This difference can be attributed to the presence of only a single electrostatic anchoring point in the **CP5A:Z3** complex, thus resulting in a lesser degree of immobilization of the guest.

Finally, to further validate the performance of the zwitterionic pillar[5]­arene under high-salinity conditions, a calorimetric evaluation of **ZP5A** with **Z3** is conducted in the presence of an excess of an external salt (Figure S37). In this case, NaBF_4_ is used at a concentration 100 times greater than that of the macrocycle. This substantial presence of BF_4_^–^, combined with its ionic radius, which is comparable to that of the cationic groups on the receptor’s portals, creates a scenario designed to mimic conditions where these external anions could easily induce screening effects, thereby diminishing the binding affinity of **ZP5A** for **Z3**. However, the calorimetric experiments yield a binding constant value of *K*_**ZP5A**:**Z3**_ = (2.4 ± 0.1) × 10^3^ M^–1^, indicating that, despite a slight decrease, the affinity of the macrocycle remains relatively stable and is not significantly affected by the high-salinity conditions employed in this analysis. This result confirms the applicability of **ZP5A** in encapsulating zwitterions in complex media, a task that is often challenging due to the high stabilization of these species in their free form in aqueous solution.

## Conclusions

This study reveals the considerable potential of zwitterionic synthetic receptors with rationally engineered architectures for achieving more efficient molecular recognition. A distinct advantage of this approach is the absence of formal counterions, which inherently prevents self-ion-pairing phenomena and provides a competitive edge over receptors that are exclusively polyanionic or polycationic. Such receptors are often prone to screening effects, especially as the host concentration increases, which can severely diminish binding affinity. In the context of supramolecular chemistry, eliminating host concentration as a controlling factor in the binding process is particularly valuable as analytical techniques used to determine binding affinities vary in sensitivity and concentration ranges, often resulting in discrepancies in calculated binding constants. Another key benefit of this synthetic strategy is the development of a zwitterionic system with permanently maintained charges, irrespective of pH, where charge centers exhibit strong mutual affinity. Facilitated by the receptor’s structural flexibility, this attraction prevents external ions from outcompeting for the recognition sites, thereby supporting reliable performance even in high-salinity environments that simulate physiological conditions. In such settings, intramolecular stabilization arising from within/between the zwitterionic functionalities keeps the receptor’s supramolecular binding fairly intact, making these systems especially promising for applications in complex biological scenarios where stable host–guest interactions are essential. Additionally, synergistic interplay with zwitterionic guests brings further advantages to this design, given the inherent challenges of complexing these molecules due to their strong solvation in aqueous solution. These developments hold significant potential, unlocking new opportunities for the application of supramolecular hosts not only in clinical and therapeutic contexts but also in salt-rich environments, where molecular interactions are typically limited.

## Supplementary Material



## Data Availability

The data underlying this study are available in the published article and its Supporting Information.

## Notes

Experimental methods (synthesis and characterization), determination of self-diffusion coefficients, calculation of thermodynamic parameters, and assessment of host–guest interactions (PDF)

## References

[1] Sehgal V., Pandey S. P., Singh P. K. (2024). Prospects of Charged Cyclodextrins
in Biomedical Applications. Carbohydr. Polym..

[2] Yin H., Cheng Q., Bardelang D., Wang R. (2023). Challenges and Opportunities
of Functionalized Cucurbiturils for Biomedical Applications. JACS Au.

[3] Pan Y.-C., Hu X.-Y., Guo D.-S. (2021). Biomedical
Applications of Calixarenes:
State of the Art and Perspectives. Angew. Chem.,
Int. Ed..

[4] Zhu H., Li Q., Khalil-Cruz L. E., Khashab N. M., Yu G., Huang F. (2021). Pillararene-Based Supramolecular Systems for Theranostics and Bioapplications. Sci. China Chem..

[5] Li X., Shen M., Yang J., Liu L., Yang Y.-W. (2024). Pillararene-Based
Stimuli-Responsive Supramolecular Delivery Systems for Cancer Therapy. Adv. Mater..

[6] Gómez-González B., Basílio N., Vaz B., Góñez K. V., Pérez-Lorenzo M., García-Río L. (2024). Supramolecular Engineering
of Micellar Systems: Precision Control on Self-Assembly, Polarity,
and Charge for Enhanced Nanocarrier Design. J. Mol. Liq..

[7] Sarkar S., Chatterjee A., Kim D., Saritha C., Barman S., Jana B., Ryu J.-H., Das A. (2025). Host–Guest
Adduct
as a Stimuli-Responsive Prodrug: Enzyme-Triggered Self-Assembly Process
of a Short Peptide Within Mitochondria to Induce Cell Apoptosis. Adv. Healthc. Mater..

[8] Saji V. S. (2022). Recent
Updates on Supramolecular-Based Drug Delivery – Macrocycles
and Supramolecular Gels. Chem. Rec..

[9] Zhou X., Bai X., Zhang X., Wu J., Liu Y. (2024). Cucurbit­[8]­uril Induced
Molecular Folding Cascade Assembly for NIR Targeted Cell Imaging. Adv. Opt. Mater..

[10] Geng W.-C., Ye Z., Zheng Z., Gao J., Li J.-J., Shah M. R., Xiao L., Guo D.-S. (2021). Supramolecular Bioimaging through
Signal Amplification by Combining Indicator Displacement Assay with
Förster Resonance Energy Transfer. Angew.
Chem., Int. Ed..

[11] Gnaim S., Scomparin A., Eldar-Boock A., Bauer C. R., Satchi-Fainaro R., Shabat D. (2019). Light Emission Enhancement by Supramolecular Complexation
of Chemiluminescence Probes Designed for Bioimaging. Chem. Sci..

[12] Tian J.-H., Xu H., Hu X.-Y., Guo D.-S. (2024). Supramolecular Fluorescence Biosensing
Based on Macrocycles. Supramol. Mater..

[13] Chen J., Tabaie E. Z., Hickey B. L., Gao Z., Raz A. A. P., Li Z., Wilson E. H., Hooley R. J., Zhong W. (2023). Selective
Molecular Recognition and Indicator Displacement Sensing of Neurotransmitters
in Cellular Environments. ACS Sensors.

[14] Zhong W., Hooley R. J. (2022). Combining Excellent Selectivity with Broad Target Scope:
Biosensing with Arrayed Deep Cavitand Hosts. Acc. Chem. Res..

[15] Wu D., Wang J., Du X., Cao Y., Ping K., Liu D. (2024). Cucurbit­[8]­uril-Based Supramolecular
Theranostics. J. Nanobiotechnology.

[16] Zhou J., Rao L., Yu G., Cook T. R., Chen X., Huang F. (2021). Supramolecular
Cancer Nanotheranostics. Chem. Soc. Rev..

[17] Yu G., Chen X. (2019). Host-Guest Chemistry
in Supramolecular Theranostics. Theranostics.

[18] Maity D. (2023). Recent Advances
in the Modulation of Amyloid Protein Aggregation Using the Supramolecular
Host-Guest Approaches. Biophys. Chem..

[19] Quan J., Zhang X., Ding Y., Li S., Qiu Y., Wang R., Zhou X. (2021). Cucurbit­[7]­uril as
a Broad-Spectrum
Antiviral Agent against Diverse RNA Viruses. Virol. Sin..

[20] Gao L., Wang H., Zheng B., Huang F. (2021). Combating Antibiotic
Resistance: Current Strategies for the Discovery of Novel Antibacterial
Materials Based on Macrocycle Supramolecular Chemistry. Giant.

[21] Li X., Bai H., Yang Y., Yoon J., Wang S., Zhang X. (2019). Supramolecular
Antibacterial Materials for Combatting Antibiotic Resistance. Adv. Mater..

[22] Liu H., Lv J., Wang X., Dong S., Li X., Gao L. (2024). Construction
of a Supramolecular Antibacterial Material Based on Water-Soluble
Pillar[5]­arene and a Zwitterionic Guest Molecule. Chem. Commun..

[23] Brockett A. T., Xue W., King D., Deng C.-L., Zhai C., Shuster M., Rastogi S., Briken V., Roesch M. R., Isaacs L. (2023). Pillar­[6]­MaxQ:
A Potent Supramolecular Host for in Vivo Sequestration of Methamphetamine
and Fentanyl. Chem..

[24] Liu H.-K., Lin F., Yu S.-B., Wu Y., Lu S., Liu Y.-Y., Qi Q.-Y., Cao J., Zhou W., Li X., Wang H., Zhang D.-W., Li Z.-T., Ma D. (2022). Highly Water-Soluble
Cucurbit[8]­uril Derivative as a Broad-Spectrum Neuromuscular Block
Reversal Agent. J. Med. Chem..

[25] Deng C.-L., Murkli S. L., Isaacs L. D. (2020). Supramolecular Hosts as in Vivo Sequestration
Agents for Pharmaceuticals and Toxins. Chem.
Soc. Rev..

[26] Liu, Y. ; Shi, D. ; Li, B. ; Jin, Y. ; Ling, D. ; Li, F. Supramolecular Macrocyclic Artificial Ion Channels for Biomedical Applications. Fundam. Res. 2024, 10.1016/j.fmre.2024.06.012. PMC12848136 41613438

[27] Tu Y., Peng F., Adawy A., Men Y., Abdelmohsen L. K. E. A., Wilson D. A. (2016). Mimicking the Cell:
Bio-Inspired Functions of Supramolecular
Assemblies. Chem. Rev..

[28] Zhao Y., Song S., Ren X., Zhang J., Lin Q., Zhao Y. (2022). Supramolecular Adhesive
Hydrogels for Tissue Engineering Applications. Chem. Rev..

[29] Wang S., Ong P. J., Liu S., Thitsartarn W., Tan M. J. B. H., Suwardi A., Zhu Q., Loh X. J. (2022). Recent
Advances in Host-Guest Supramolecular Hydrogels for Biomedical Applications. Chem.—Asian J..

[30] Escobar L., Ballester P. (2021). Molecular Recognition in Water Using Macrocyclic Synthetic
Receptors. Chem. Rev..

[31] Biedermann F. (2019). Water-Compatible
Host Systems. Supramol. Chem. Water.

[32] Chen F.-Y., Geng W.-C., Cai K., Guo D.-S. (2024). Molecular
Recognition
of Cyclophanes in Water. Chin. Chem. Lett..

[33] Beatty M. A., Hof F. (2021). Host–Guest Binding in Water, Salty Water, and Biofluids: General
Lessons for Synthetic, Bio-Targeted Molecular Recognition. Chem. Soc. Rev..

[34] Strutt N. L., Zhang H., Schneebeli S. T., Stoddart J. F. (2014). Functionalizing
Pillar­[n]­arenes. Acc. Chem. Res..

[35] Yang W., Zhang W., Chen J., Zhou J. (2024). Mono-Functionalized
Pillar­[n]­arenes: Syntheses, Host–Guest Properties and Applications. Chin. Chem. Lett..

[36] Han C., Zhao D., Lü Z., Zhan F., Zhang L., Dong S., Jin L. (2019). Synthesis
of a Difunctionalized Pillar[5]­arene
with Hydroxyl and Amino Groups at A1/A2 Positions. Eur. J. Org. Chem..

[37] Hu W.-B., Hu W.-J., Zhao X.-L., Liu Y. A., Li J.-S., Jiang B., Wen K. (2016). A1/A2-Diamino-Substituted
Pillar­[5]­arene-Based
Acid–Base-Responsive Host–Guest System. J. Org. Chem..

[38] Zeng H., Liu P., Xing H., Huang F. (2022). Symmetrically Tetra-Functionalized
Pillar[6]­arenes Prepared by Fragment Coupling. Angew. Chem., Int. Ed..

[39] Ogoshi T., Yamafuji D., Kotera D., Aoki T., Fujinami S., Yamagishi T. (2012). Clickable
Di- and Tetrafunctionalized Pillar­[n]­arenes
(n = 5, 6) by Oxidation–Reduction of Pillar­[n]­arene Units. J. Org. Chem..

[40] Wu L., Han C., Jing X., Yao Y. (2021). Rim-Differentiated Pillar[5]­arenes. Chin. Chem.
Lett..

[41] Demay-Drouhard P., Du K., Samanta K., Wan X., Yang W., Srinivasan R., Sue A. C.-H., Zuilhof H. (2019). Functionalization
at Will of Rim-Differentiated
Pillar[5]­arenes. Org. Lett..

[42] Bleus S., Dehaen W. (2024). Pillararene-Inspired
Arenes: Synthesis, Properties
and Applications Compared to the Parent Macrocycle. Coord. Chem. Rev..

[43] Fu S., An G., Sun H., Luo Q., Hou C., Xu J., Dong Z., Liu J. (2017). Laterally Functionalized Pillar[5]­arene:
A New Building Block for Covalent Self-Assembly. Chem. Commun..

[44] Wang Z., Liu Y. A., Yang H., Hu W.-B., Wen K. (2022). Ortho-Functionalization
of Pillar[5]­arene: An Approach to Mono-Ortho-Alkyl/Aryl-Substituted
A1/A2-Dihydroxypillar[5]­arene. Org. Lett..

[45] Strutt N. L., Zhang H., Schneebeli S. T., Stoddart J. F. (2014). Amino-Functionalized
Pillar­[5]­arene. Chem.—Eur. J..

[46] Kubik S. (2022). When Molecules
Meet in Water-Recent Contributions of Supramolecular Chemistry to
the Understanding of Molecular Recognition Processes in Water. ChemistryOpen.

[47] Gómez-González B., Basílio N., Vaz B., Pérez-Lorenzo M., García-Río L. (2024). Delving into the Variability of Supramolecular
Affinity: Self-Ion Pairing as a Central Player in Aqueous Host-Guest
Chemistry. Angew. Chem., Int. Ed..

[48] Gómez B., Francisco V., Fernández-Nieto F., Garcia-Rio L., Martín-Pastor M., Paleo M. R., Sardina F. J. (2014). Host–Guest
Chemistry of a Water-Soluble Pillar[5]­arene: Evidence for an Ionic-Exchange
Recognition Process and Different Complexation Modes. Chem.—Eur. J..

[49] Francisco V., Basilio N., García-Río L. (2012). Counterion Exchange
as a Decisive Factor in the Formation of Host:Guest Complexes by p-Sulfonatocalix[4]­arene. J. Phys. Chem. B.

[50] Jordan J. H., Ashbaugh H. S., Mague J. T., Gibb B. C. (2021). Buffer and Salt
Effects in Aqueous Host–Guest Systems: Screening, Competitive
Binding, or Both?. J. Am. Chem. Soc..

[51] Francisco V., Piñeiro A., Nau W. M., García-Río L. (2013). The “True”
Affinities of Metal Cations to p-Sulfonatocalix[4]­arene: A Thermodynamic
Study at Neutral pH Reveals a Pitfall Due to Salt Effects in Microcalorimetry. Chem.—Eur. J..

[52] Gasa T. B., Valente C., Stoddart J. F. (2011). Solution-Phase Counterion Effects
in Supramolecular and Mechanostereochemical Systems. Chem. Soc. Rev..

[53] Lin Q., Ding X., Hou Y., Ali W., Li Z., Han X., Meng Z., Sun Y., Liu Y. (2024). Adsorption and Separation
Technologies Based on Supramolecular Macrocycles for Water Treatment. Eco-Environment Heal..

[54] Li Z., Kang W., Yang H., Zhou B., Jiang H., Liu D., Jia H., Wang J. (2022). Advances of Supramolecular Interaction
Systems for Improved Oil Recovery (IOR). Adv.
Colloid Interface Sci..

[55] Abdelhay A. H., Bani-Yaseen A. D. (2024). Recent Advances and Perspectives
of Supramolecular
Host-Guest Systems for Electrochemical Energy Storage. Mater. Today Chem..

[56] Lou X.-Y., Zhang S., Wang Y., Yang Y.-W. (2023). Smart Organic
Materials
Based on Macrocycle Hosts. Chem. Soc. Rev..

[57] Gaohua L., Miao X., Dou L. (2021). Crosstalk
of Physiological pH and
Chemical pK_a_ under the Umbrella of Physiologically Based
Pharmacokinetic Modeling of Drug Absorption, Distribution, Metabolism,
Excretion, and Toxicity. Expert Opin. Drug Metab.
Toxicol..

[58] Shurpik D. N., Sevastyanov D. A., Zelenikhin P. V., Padnya P. L., Evtugyn V. G., Osin Y. N., Stoikov I. I. (2020). Nanoparticles
Based on the Zwitterionic
Pillar[5]­arene and Ag^+^: Synthesis, Self-Assembly and Cytotoxicity
in the Human Lung Cancer Cell Line A549. Beilstein
J. Nanotechnol..

[59] Collins K. D. (2019). The Behavior
of Ions in Water Is Controlled by Their Water Affinity. Q. Rev. Biophys..

[60] Cohen Y., Slovak S., Avram L. (2021). Solution NMR of Synthetic
Cavity
Containing Supramolecular Systems: What Have We Learned on and From?. Chem. Commun..

[61] Archer W. R., Schulz M. D. (2020). Isothermal Titration
Calorimetry: Practical Approaches
and Current Applications in Soft Matter. Soft
Matter.

[62] Suman S., Singh R. (2019). Anion Selective Electrodes: A Brief Compilation. Microchem. J..

[63] Alfonso I., Solà J. (2020). Molecular Recognition of Zwitterions
with Artificial
Receptors. Chem.—Asian J..

[64] Chaudhary K. N., Brosnahan K. I., Gibson-Elias L. J., Moreno J. L., Hickey B. L., Hooley R. J., Caulkins B. G. (2024). Investigation of the Effects on Proton
Relaxation Times upon Encapsulation in a Water-Soluble Synthetic Receptor. Phys. Chem. Chem. Phys..

