# Supplementary material for: Rational Design of Supramolecular Receptors for Consistent Binding Affinities under High-Salinity Conditions
Source: J Org Chem. 2025 Apr 17;90(18):6134–45. doi: 10.1021/acs.joc.5c00068 (PMC12133026; doi:10.1021/acs.joc.5c00068)
Supplement: Supplementary file 1 [file jo5c00068_si_001.pdf]

Supporting Information

Rational Design of Supramolecular Receptors for Consistent Binding Affinities under High-Salinity Conditions

Borja Gómez-González,[a] *Nuno Basílio,*[b] *Belén Vaz,*[c,d] *M. Rita Paleo,*[e,f] *F. Javier Sardina,*[f,g] *Moisés Pérez-Lorenzo,* *[c,d] *and Luis García-Río* *[a,g]

[a] Department of Physical Chemistry, Universidade de Santiago de Compostela, 15782 Santiago de Compostela, Spain.

[b] Laboratório Associado para a Química Verde (LAQV), Rede de Química e Tecnologia (REQUIMTE), Departamento de Química, Faculdade de Ciências e Tecnologia, Universidade NOVA de Lisboa, 2829-516 Caparica, Portugal.

[c] CINBIO, Universidade de Vigo, 36310 Vigo, Spain.

[d] Galicia Sur Health Research Institute, 36310 Vigo, Spain.

[e] Center for Research in Biological Chemistry and Molecular Materials (CIQUS), Universidade de Santiago de Compostela, 15782 Santiago de Compostela, Spain.

[f] Department of Organic Chemistry, Universidade de Santiago de Compostela, 15782 Santiago de Compostela, Spain.

[g] Centro de Investigación Mestrelab (CIM), Av. Barcelona 7, Santiago de Compostela 15706, Spain.

*Email: moisespl@uvigo.es; luis.garcia@usc.es

INDEX

Section 1. Experimental methods S2

Section 2. Determination of self-diffusion coefficients S23

Section 3. Determination of thermodynamic parameters for anionic guests S27

Section 4. Characterization of host-guest interactions S30

Section 5. Determination of thermodynamic parameters for zwitterionic guests S32

Section 6. References S35

Section 1. Experimental methods

1. Reagents

All chemicals were of analytical grade and used without further purification. **ZP5A** and **Z3** were synthesized in-house. Milli-Q water (18.2 MΩ cm^{-1}) was used in all the preparations.

2. Characterization

2.1. Nuclear magnetic resonance spectroscopy (NMR)

^{1}H and ^{13}C NMR spectra were recorded in deuterated solvent ($CDCl_3$, CD_3OD or D_2O at 25.0 ºC on a Varian Mercury INOVA 300 NMR spectrometer, operating at 300 MHz for ^{1}H and 75 MHz for ^{13}C. Chemical shifts (δ) are given in parts per million (ppm), and coupling constants (*J*) are given in hertz (Hz). The residual protic solvent is taken as internal reference. The proton spectra are reported as follows: δ (multiplicity, coupling constant *J*, number of protons) with residual protic solvent as the internal reference: $CDCl_3$, δ = 7.26 ppm; D_2O, δ = 4.80 ppm; CD_3OD, δ = 3.31 ppm for ^{1}H NMR and $CDCl_3$ (δ_C = 77.2 ppm), and CD_3OD (δ_C = 49.0 ppm) for ^{13}C NMR. 2D NMR spectra were recorded on a Varian INOVA 500 NMR spectrometer. Structural assignments were made with additional information from gCOSY, gHSQC, and gHMBC experiments. NMR spectra were processed using Mnova 14.3.3.

2.2. Diffusion-ordered spectroscopy (DOSY)

The DOSY bipolar pulse pair stimulated echo (DBPPSTE) sequence was employed to determine the self-diffusion coefficients. Rectangular-shaped pulse field gradients, 1.6 ms in duration, were applied with linearly incremented strength levels ranging from 2.1 to 64.3 Gcm^{-1} in 20 steps. To calibrate the gradient strengths, the diffusion coefficient of residual HDO in a 1%/99% H_2O/D_2O sample at 25.0 °C was set to 1.872×10^{-9} m^2s^{-1}. To ensure accurate measurements of the self-diffusion coefficients, the diffusion time was optimized for each sample in order to smoothly capture the signal attenuation and to maximize the intensity difference between the traces with the maximum and minimum gradient strengths. The experimental data were fitted to Equation [1]:

$$I = I_0 e^{-\gamma^2 \delta^2 G^2 \left(\Delta - \frac{\delta}{3}\right) D} = I_0 e^{-bD} \qquad [1]$$

where *I* is the observed intensity, I_0 is the reference intensity (unattenuated signal intensity), *γ* is the gyromagnetic ratio of the observed nucleus, *G* is the gradient strength, δ is the length of the pulsed field gradient, Δ is the diffusion time, and *D* is the diffusion coefficient. Using this equation, *D* was obtained by performing a nonlinear fit of the intensity decay data as a function of *b*.

2.3. Electrospray ionization mass spectrometry (ESI-MS)

High resolution ESI-MS spectra were obtained on a Bruker Amazon ETD spectrometer with ion trap analyzer, in positive ion mode.

2.4. Thermogravimetry (TGA)

Thermogravimetric analysis was conducted in duplicate on 4.5 mg samples of **ZP5A** using a TGA Q5000 analyzer (TA Instruments).

2.5. Ion-selective electrode potentiometry (ISE)

Free bromide ion concentrations in the **ZP5A** aqueous solutions were measured at 25.0 ºC on an ion-selective electrode (Mettler Toledo, DX280-Br).

2.6. Isothermal Titration Calorimetry (ITC)

ITC measurements were conducted at 25.0 ºC using a MicroCal™ VP-ITC isothermal titration calorimeter (MicroCal, Inc.). Before each experiment, samples were degassed with a ThermoVac vacuum pump (MicroCal, Inc.). In a typical setup, 8.0–10.0 µL of a guest solution (7.0–15.0 mM) was injected every 2 min, totaling 28 to 35 injections, into a host sample (1.459 mL, 0.1–0.5 mM) under continuous stirring (459 rpm) to ensure homogeneous conditions. For each experiment, the initial injection was excluded from data analysis. Control experiments determined the heat contributions from the dilution of **ZP5A**, **CP5A**, and individual guests. The net enthalpy of the binding process was calculated by subtracting the dilution heat contributions of the host and guest. Baseline corrections were performed using the software Origin (OriginLab Corporation). Data were exported and fitted in the software AFFINImeter (Software for Science Developments (S4Sd)).

3. Synthetic procedures

All reactions involving reagents sensitive to oxygen or moisture were carried out under an argon atmosphere in glassware previously dried in the oven at 150 ºC for at least 12 h, and flamed dry under dry argon atmosphere.

3.1. Synthesis of ZP5A

The synthetic strategy for the preparation of the pillararene endowed with zwitterionic functionalities is outlined in Scheme S-1.

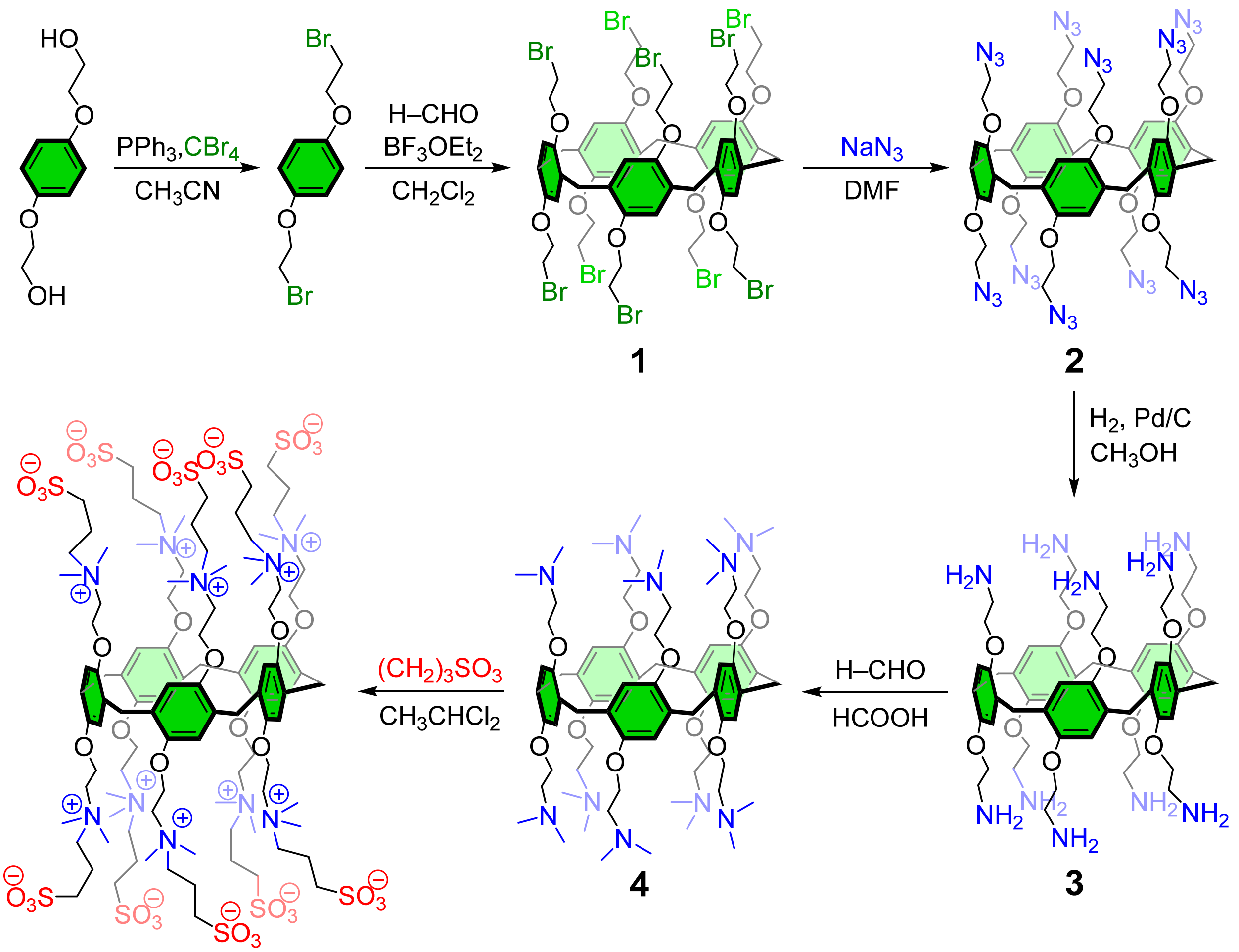

Scheme S-1. Synthetic strategy for the preparation of **ZP5A**.

3.1.1. Synthesis of 1

10-Br-pillar[5]arene **1** was synthesized as represented in Scheme S-1, according to previously reported methods.[1,2] Briefly, to a cold (0 ºC) solution of 1,4-bis(2-hydroxyethoxy)benzene (10.0 g, 50.5 mmol) and triphenylphosphine (31.5 g, 120 mmol) in dry acetonitrile (250 mL), carbon tetrabromide (39.8 g, 120 mmol) was added in portions. The resulting mixture was stirred for 4 h

at room temperature under argon. Then, cold water (200 mL) was added to the reaction mixture and a white precipitate was formed. The solid was collected by vacuum filtration, thoroughly washed with 60:40 MeOH/H_2O, and dried at high vacuum, affording 14.4 g (88%) of the expected 1,4-bis(2-bromoethoxy)benzene. Spectroscopy data matched previously reported data.[1,2]

^{1}H NMR ($CDCl_3$, 300 MHz): δ 6.86 (s, 4H); 4.25 (t, *J* = 6.3, 4H); 3.61 (t, *J* = 6.3, 4H) ppm.
^{13}C{1H} NMR ($CDCl_3$, 75 MHz): δ 152.9 (C, 2×); 116.2 (CH, 4×); 68.8 (CH_2, 2×); 29.4 (CH_2, 2×) ppm.

To a solution of 1,4-bis(2-bromoethoxy)benzene (5 g, 15.43 mmol) in dichloromethane (230 mL), paraformaldehyde (0.93 g, 30.87 mmol) was added under argon atmosphere. Then, boron trifluoride diethyl etherate ($BF_3·OEt_2$, 4.83 g, 33.86 mmol) was added and the mixture was stirred at room temperature for 2 h. Water was then added, and the layers were separated. The organic layer was washed with water (200 mL), a saturated aqueous solution of sodium bicarbonate (200 mL) and brine (200 mL). The organic layer was dried (anhydrous Na_2SO_4), and the solvent was removed under vacuum. The resulting yellow solid was dissolved in dichloromethane and stirred in the presence of activated carbon. The suspension was filtered through a pad of Celite® and concentrated to dryness under high vacuum, thus obtaining 3.37 g (73%) of a white solid identified as 10-Br-pillar[5]arene **1**. Spectroscopy data matched previously reported data.

^{1}H NMR ($CDCl_3$, 300 MHz): δ 6.92 (s, 10H); 4.23 (t, *J* = 5.7, 20H); 3.85 (s, 10H); 3.63 (t, *J* = 5.7, 20H) ppm.
^{13}C{1H} NMR ($CDCl_3$, 75 MHz): δ 149.7 (C, 10C); 129.1 (C, 10C); 116.0 (CH, 10C); 69.0 (CH_2, 10C); 30.8 (CH_2, 10C); 29.5 (CH_2, 5C) ppm.

3.1.2. Synthesis of 2

10-N_3-pillar[5]arene **2** was synthesized according to a previously reported method.[3] Sodium azide (4.8 g, 74 mmol) was added to a solution of 10-Br-pillar[5]arene **1** (2.5 g, 1.5 mmol) in dry DMF (125 mL). The resulting dispersion was stirred at 100 ºC (oil bath) under Ar atmosphere for 12 h. Once cooled to room temperature, the mixture was poured over H_2O (400 mL), and a precipitate was formed. The solid was collected by vacuum filtration, thoroughly washed with additional H_2O. After removing all the volatiles under high vacuum, 1.86 g of a white solid (95%) identified as 10-N_3-pillar[5]arene **2** was obtained. Spectroscopy data matched previously reported data.[3]

^{1}H NMR ($CDCl_3$, 300 MHz): δ 6.83 (s, 10H); 4.01 (t, *J* = 5.5, 20H); 3.84 (s, 10H); 3.55 (t, *J* = 5.5, 20H) ppm.
ESI-MS: calculated for $C_{55}H_{60}O_{10}N_{30}Na$ ($[M + Na]^+$), 1324.502; found, 1325.8986.

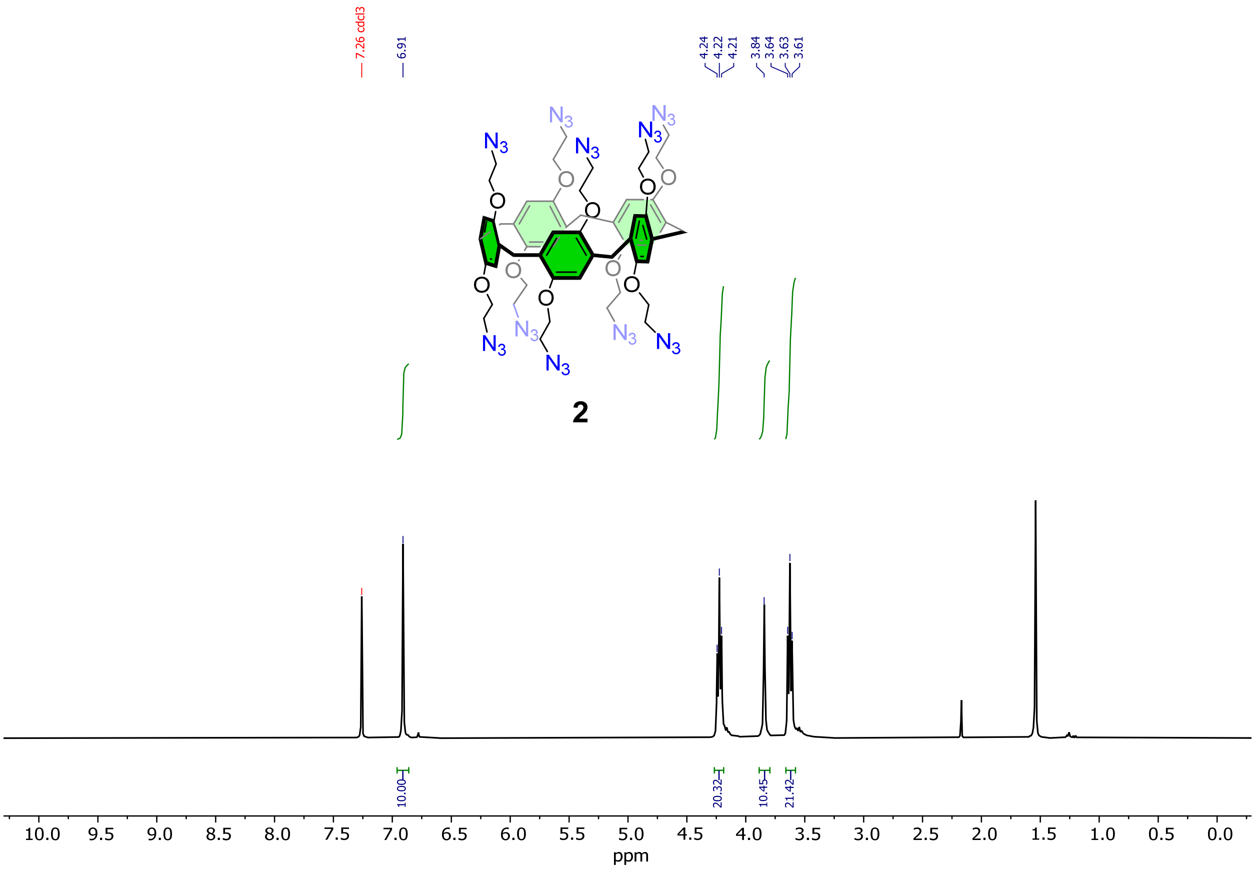

Figure S-1. ^{1}H NMR ($CDCl_3$, 300 MHz, 25.0 ºC) obtained for compound **2**.

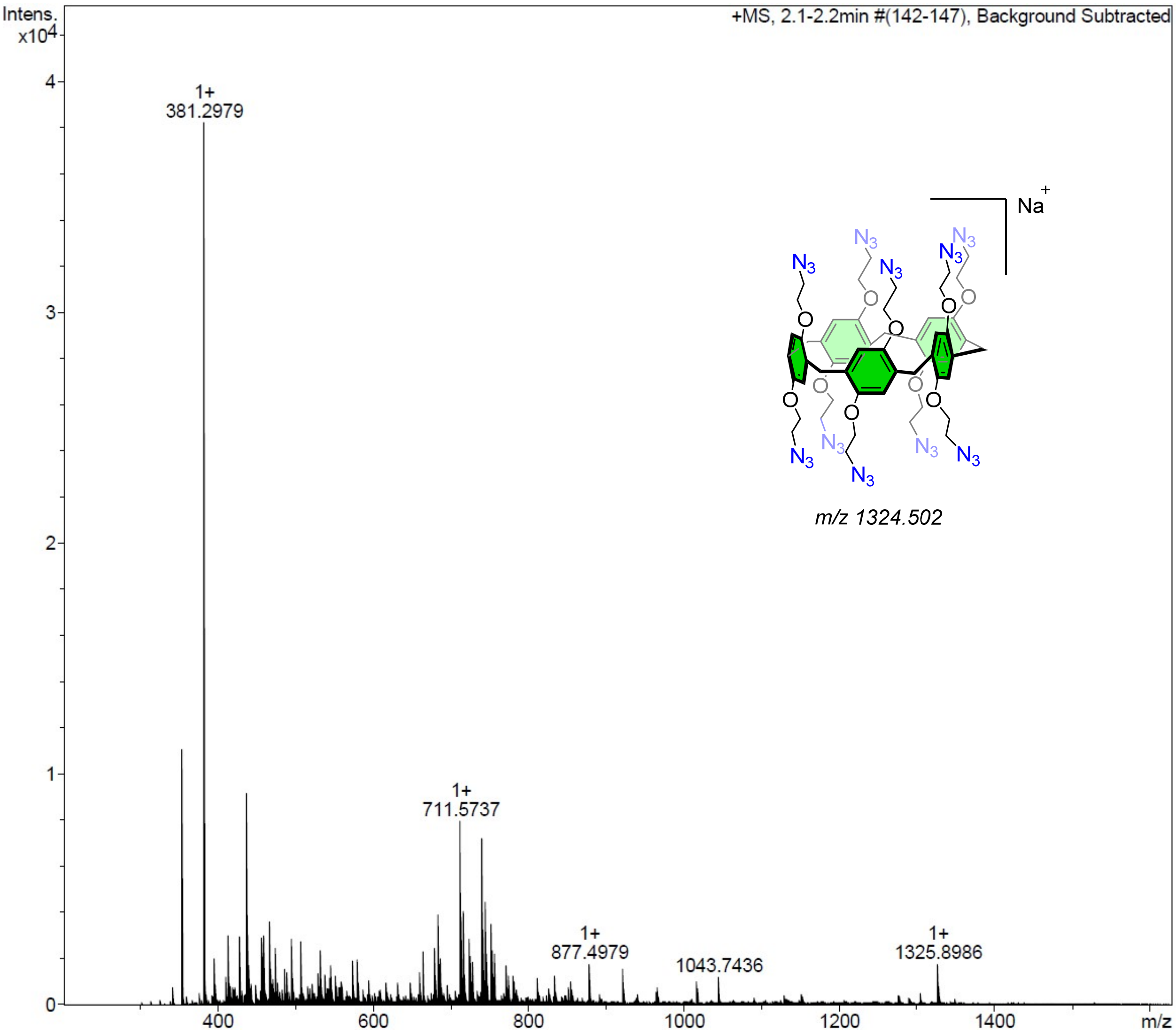

Figure S-2. ESI-MS spectrum obtained for compound **2**.

3.1.3. Synthesis of 3

Synthesis of 10-NH_2-pillar[5]arene **3** was performed according to the procedure previously described by Jun-Li Hou *et al.*[3] Pd/C (10%, 150 mg) was added to a degassed suspension of 10-N_3-pillar[5]arene **2** (1.5 g, 1.15 mmol) in methanol (90 mL). The mixture was stirred at 50 ºC (oil bath) under H_2 atmosphere for 48 h. Once cooled to room temperature, the mixture was filtered through a pad of Celite® and the resulting residue was concentrated under reduced pressure. The residue was purified by crystallization from chloroform providing 1.14 g (92%) of a white solid identified as 10-NH_2-pillar[5]arene **3**.

^{1}H NMR (CD_3OD, 300 MHz): δ 6.76z (s, 10H); 3.82 (br s, 30H); 2.93 (t, J = 5.2, 20H) ppm.
ESI-MS: calculated for $C_{55}H_{81}O_{10}N_{10}$ ($[M + H]^+$), 1041.6132; found, 1041.6133.

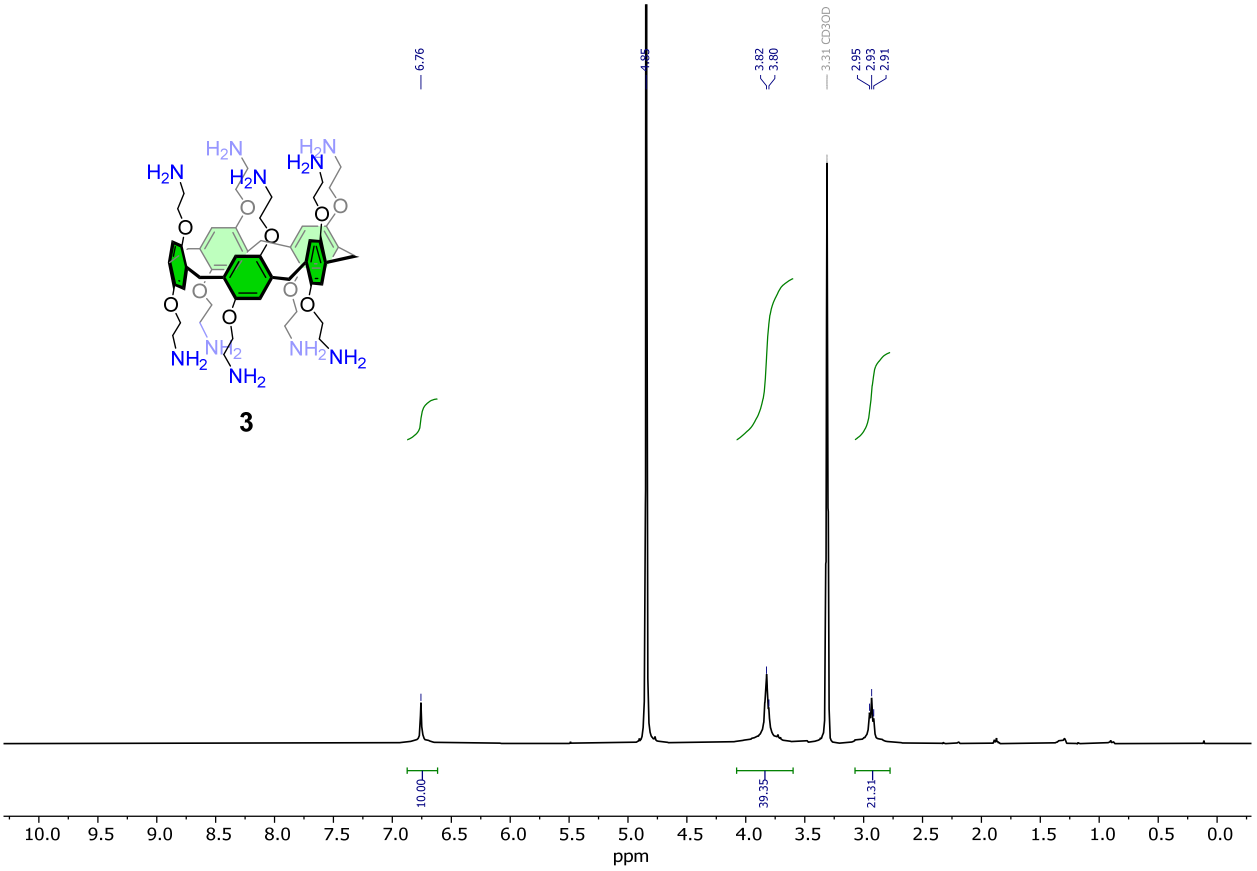

Figure S-3. ^{1}H NMR (CD_3OD, 300 MHz, 25.0 ºC) obtained for compound **3**.

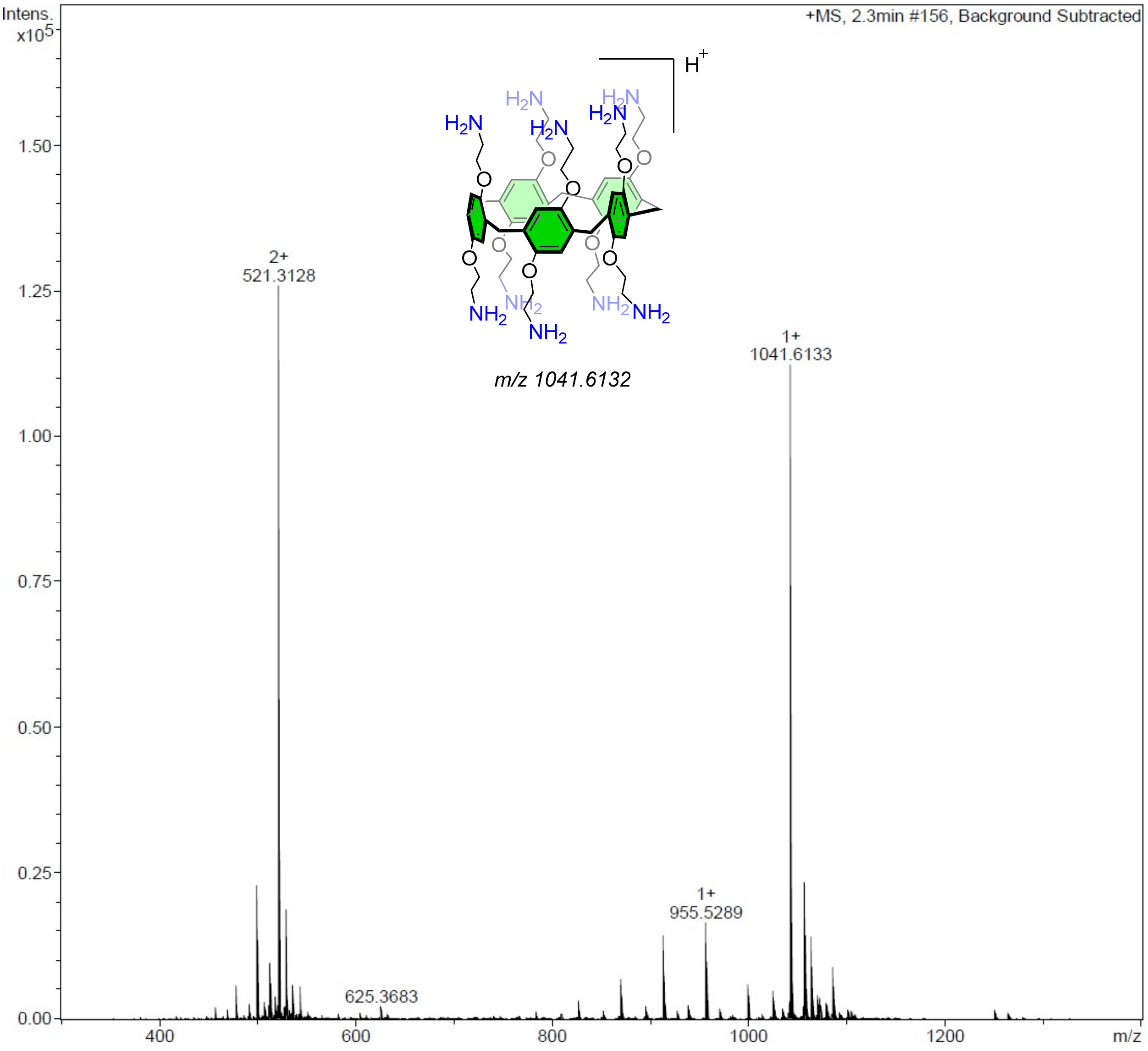

Figure S-4. ESI-MS spectrum obtained for compound **3**.

3.1.4. Synthesis of 4

Synthesis of 10-N(CH_3)$_2$-pillar[5]arene **4** was performed by an adapted method of the previously described by Alles and coworkers.[4] A flask was charged with formic acid (275 µL, 6.88 mmol). While stirring at room temperature, compound **3** (136 mg, 0.13 mmol) was added, followed by formaldehyde (37 wt. % solution in H_2O, 325 µL, 3.9 mmol). The resulting mixture was stirred in an oil bath for 16 h. Once cooled down to room temperature, the acidic mixture (pH≈3) was concentrated to dryness and redissolved with 1.0 M NaOH solution till basic pH (pH≈8). Then, the mixture was extracted with $CHCl_3$ (3×), and the combined organic layers were dried over anhydrous Na_2SO_4, filtered, and concentrated. Finally, the solid obtained was recrystallized from ethyl acetate to afford **4** as a white solid (116 mg, 67%).

^{1}H NMR (CD_3OD, 300 MHz): δ 6.87 (s, 10H); 3.98 (t, J = 5.8, 20H); 3.78 (s, 10H); 2.79 (t, J = 5.8, 20H); 2.38 (s, 60H) ppm.
ESI-MS: calculated for $C_{75}H_{121}O_{10}N_{10}$ ([M + H]$^+$), 1321.9262; found, 1321.9266.

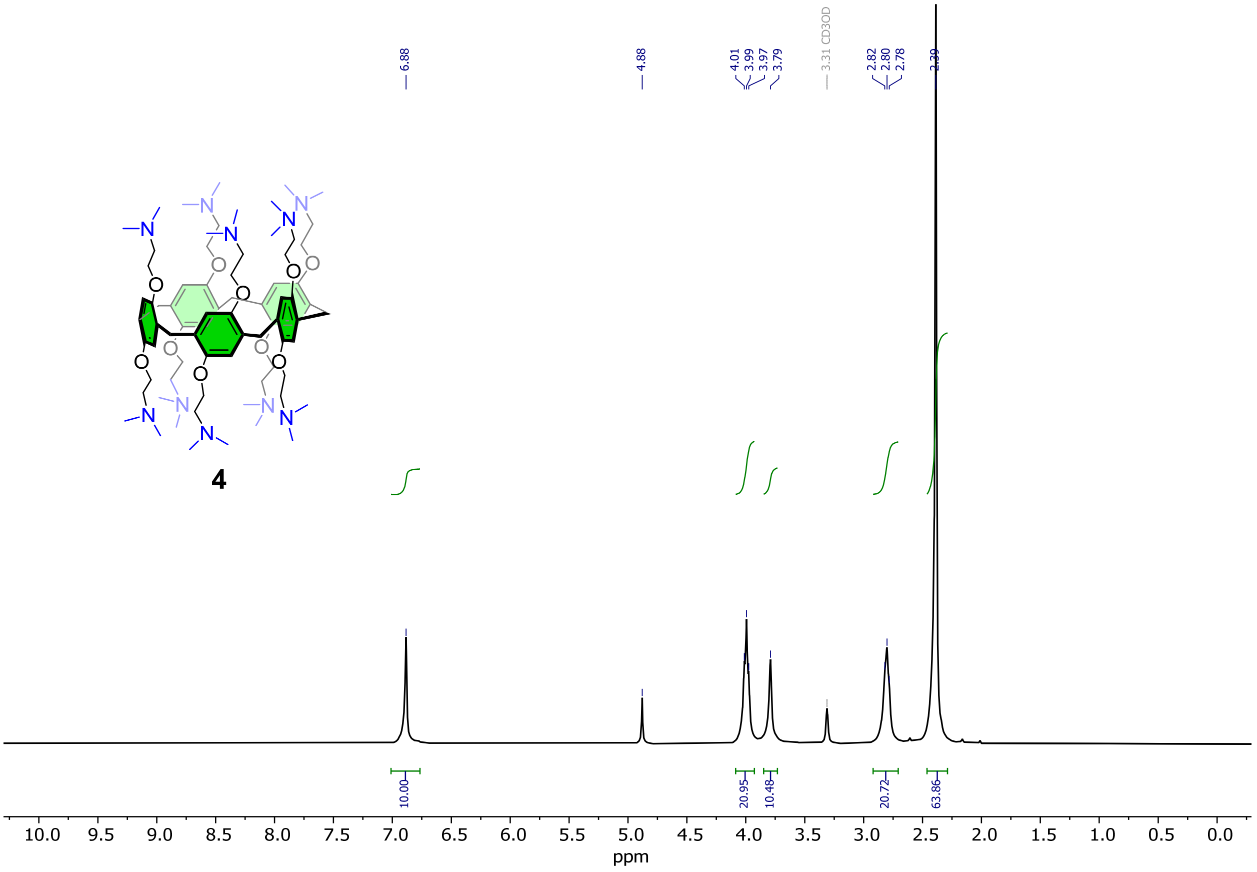

Figure S-5. ^{1}H NMR (CD_3OD, 300 MHz, 25.0 ºC) obtained for compound **4**.

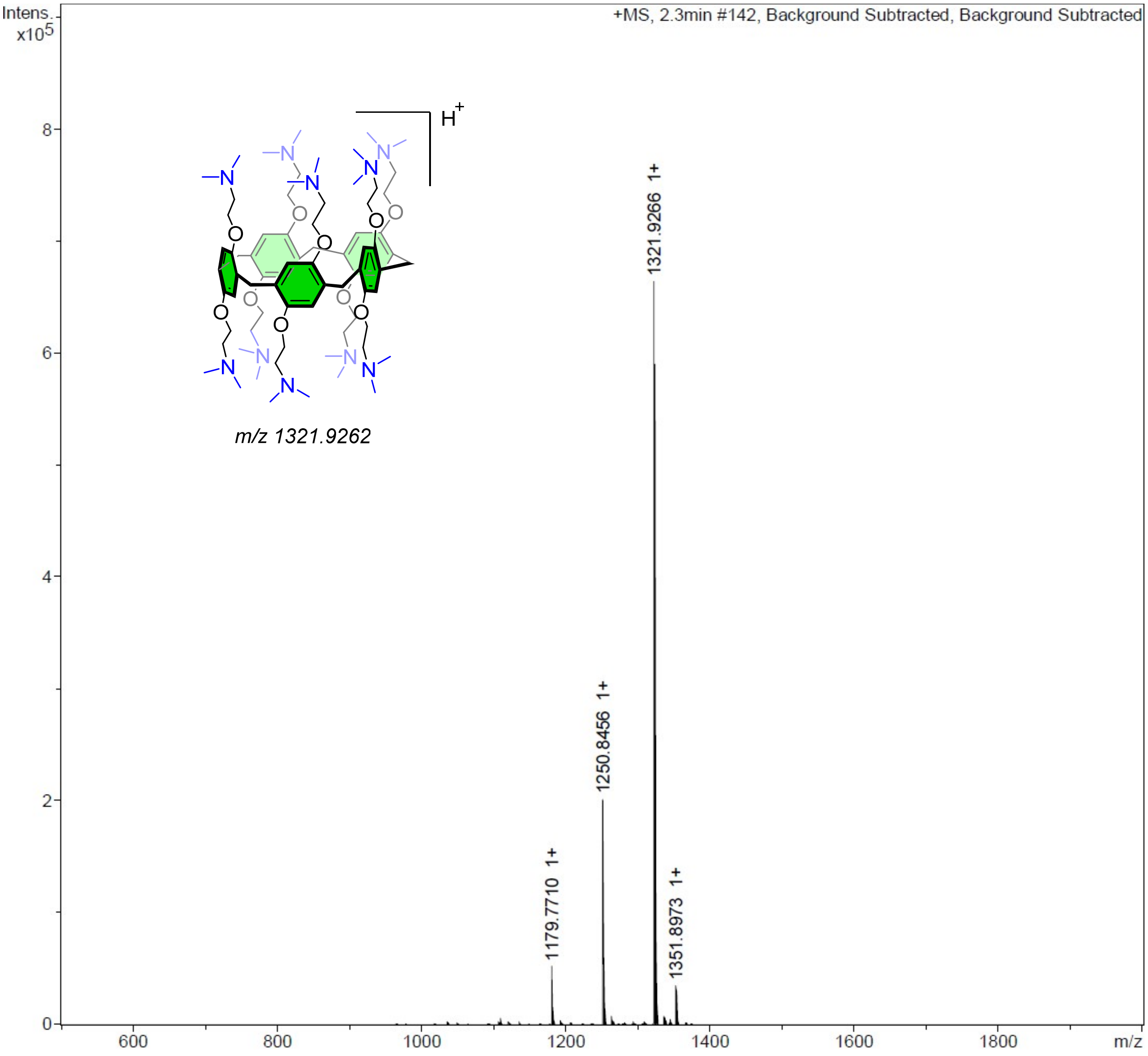

Figure S-6. ESI-MS spectrum obtained for compound **4**.

3.1.4. Synthesis of ZP5A

The procedure for the introduction of sulfopropyl groups was performed by an adapted method of the previously described by Weers *et al.*[5] To a solution of compound **4** (220 mg, 0.16 mmol) in dry dichloroethane (6.0 mL) was added 1,3-propane sultone (171 µL, 1.92 mmol). The resulting mixture was stirred at 60 °C in an oil bath for 72 h. Then, once cooled down to room temperature, the mixture was filtered under vacuum, washing the solid with CH_2Cl_2 to obtain **ZWP5** as a hygroscopic white solid (338 mg, 83%).

^{1}H NMR (D_2O, 300 MHz): δ 6.97 (br s, 10H); 4.68 – 4.41 (m, 20H); 4.08 – 3.80 (m, 30H); 3.77 – 3.61 (m, 20H); 3.29 (s, 60H); 3.05 – 2.92 (m, 20H); 2.40 – 2.18 (m, 20H) ppm.

^{13}C{1H} NMR (D_2O, 75 MHz): δ 149.0 (C, 10×), 129.2 (C, 10×), 115.7 (CH, 10×), 63.1 (CH_2, 10×), 62.7 (CH_2, 10×), 62.5 (CH_2, 10×), 51.6 (CH_3, 20×), 47.2 (CH_2, 10×), 29.3 (CH_2, 10×), 18.4 (CH_2, 10×) ppm.

ESI-MS: calculated for $C_{90}H_{151}O_{25}N_{10}S_5$ ($[M - 5(C_3H_6O_3S) + H]^+$): 1932.9480; found, 1932.9430. Calculated for $C_{87}H_{145}O_{22}N_{10}S_4$ ($[M - 6(C_3H_6O_3S) + H]^+$) 1810.9443; found, 1810.9421. Calculated for $C_{102}H_{176}O_{37}N_{10}S_9$ ($[M - (C_3H_6O_3S) + 2H]^{2+}$): 1211.4850; found, 1211.4896. Calculated for $C_{99}H_{170}O_{34}N_{10}S_8$ ($[M - 2(C_3H_6O_3S) + 2H]^{2+}$): 1149.9833; found, 1149.9859.

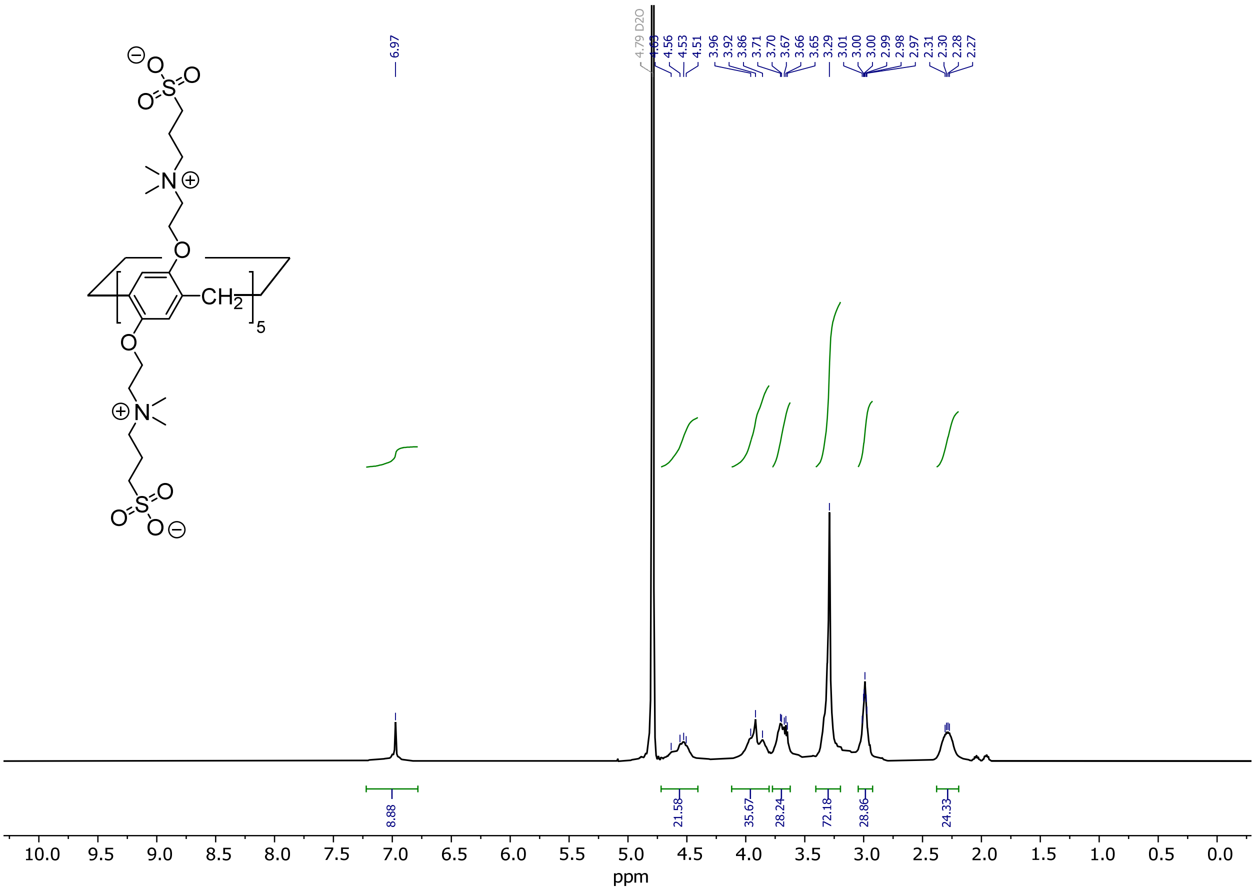

Figure S-7. ^{1}H NMR (D_2O, 500 MHz, 25.0 ºC) obtained for compound **ZP5A**.

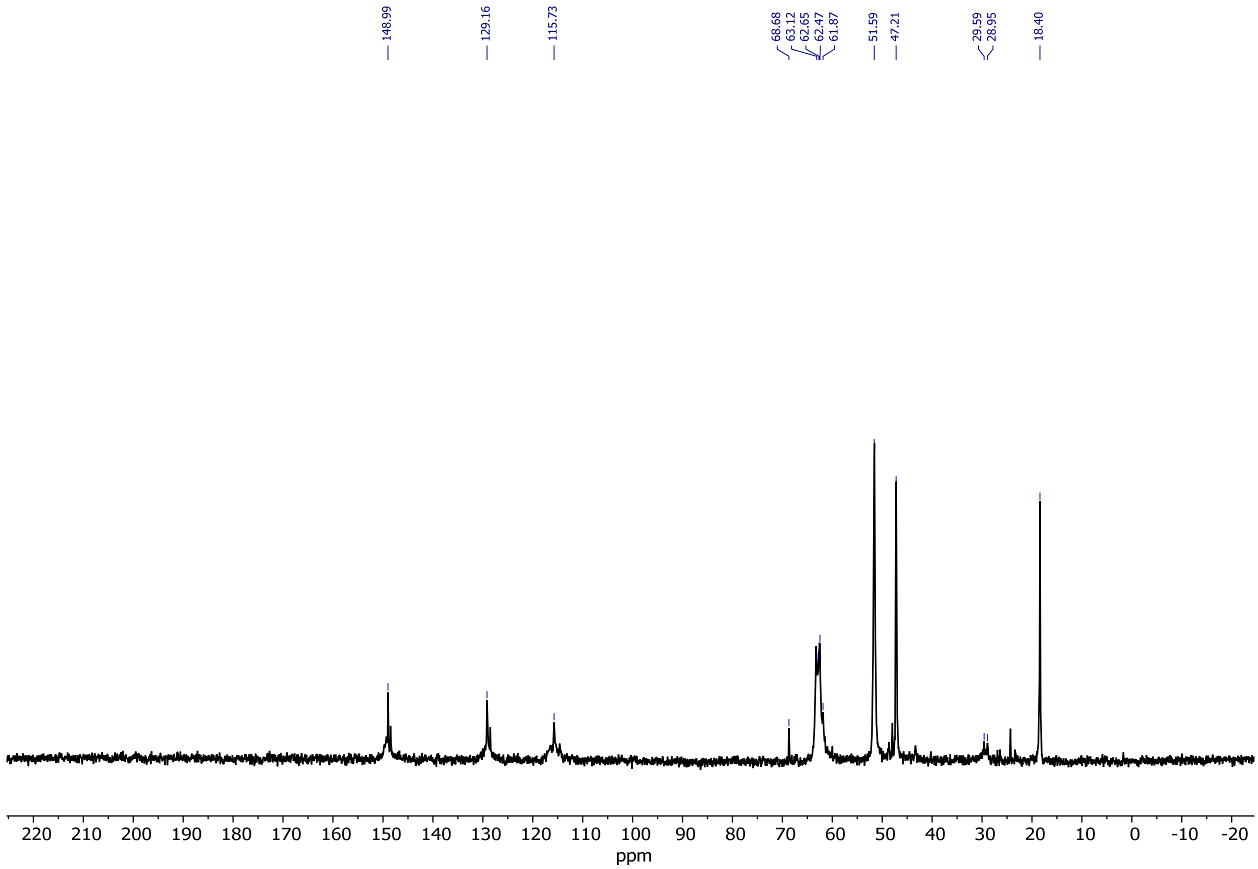

Figure S-8. $^{13}C\{1H\}$ NMR (D_2O, 500 MHz, 25.0 ºC) obtained for compound **ZP5A**.

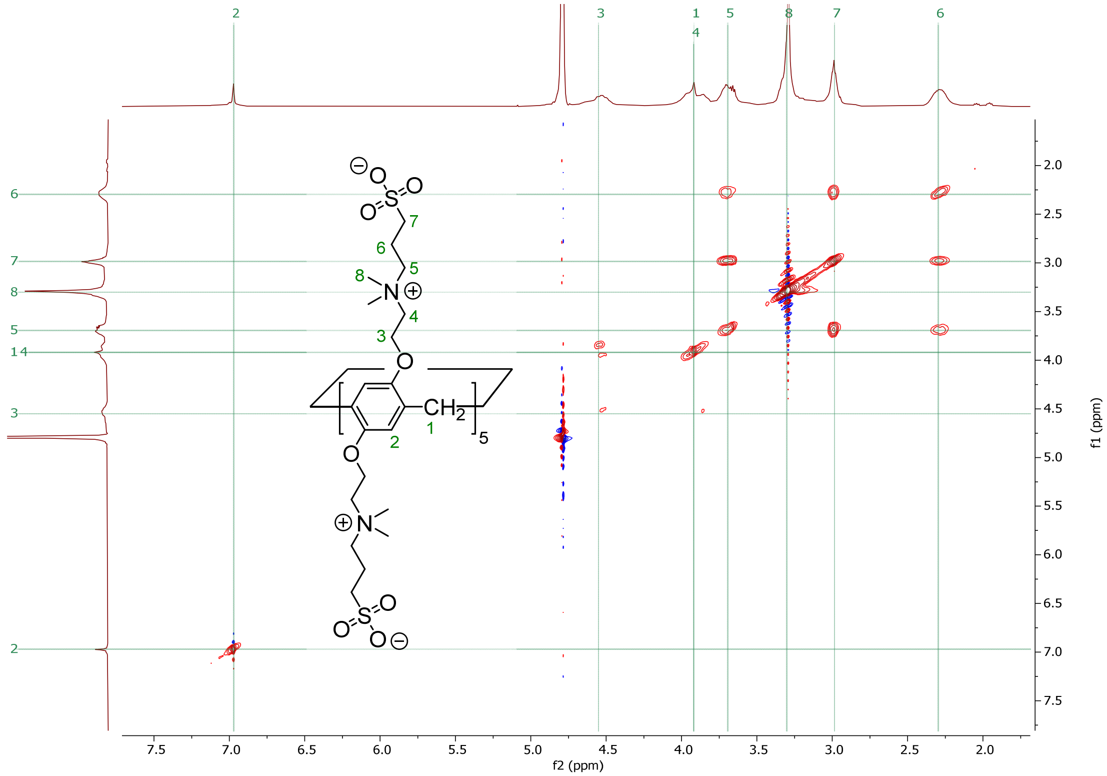

Figure S-9. TOCSY (D_2O, 500 MHz, 25.0 ºC) obtained for compound **ZP5A**.

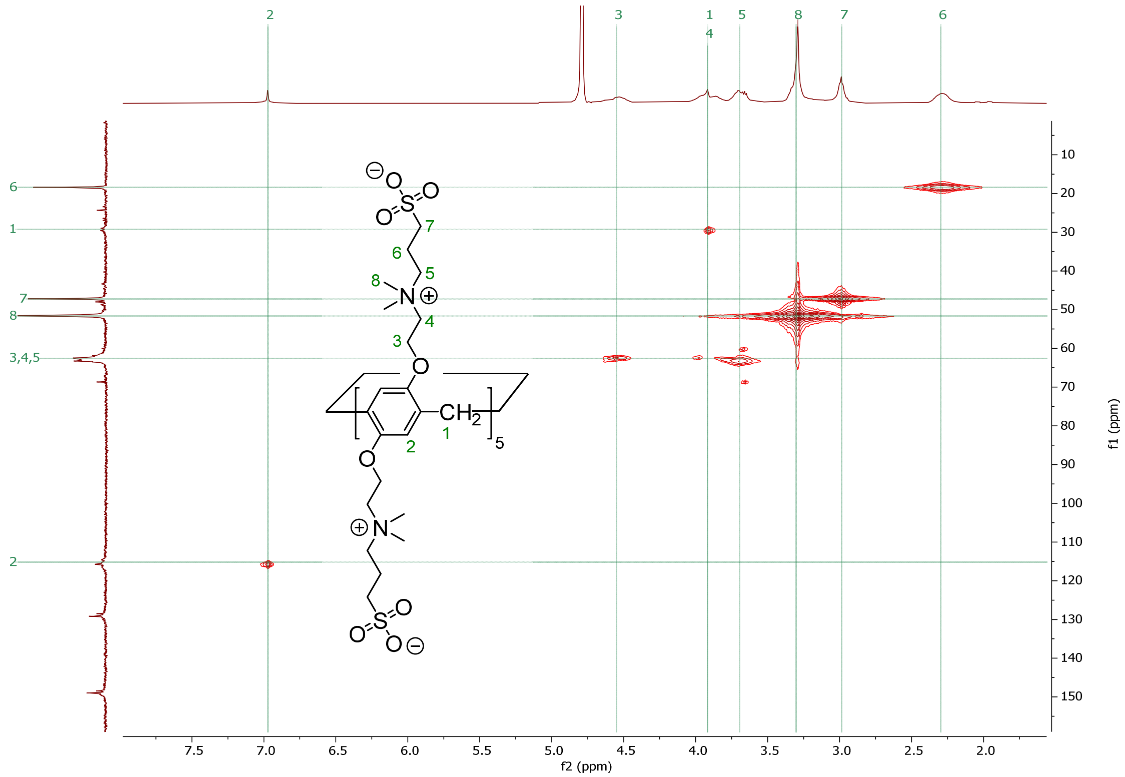

Figure S-10. HMQC (D_2O, 500;126 MHz, 25.0 ºC) obtained for compound **ZP5A**.

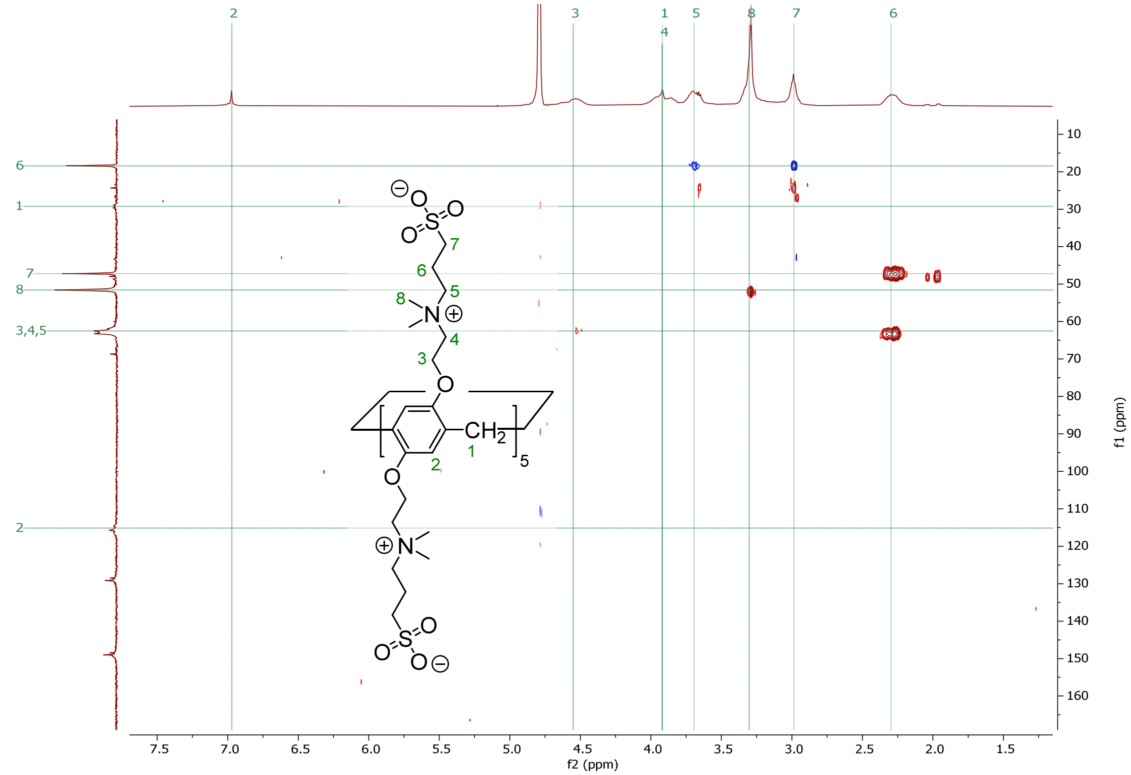

Figure S-11. H2BC (D_2O, 500;126 MHz, 25.0 ºC) obtained for compound **ZP5A**.

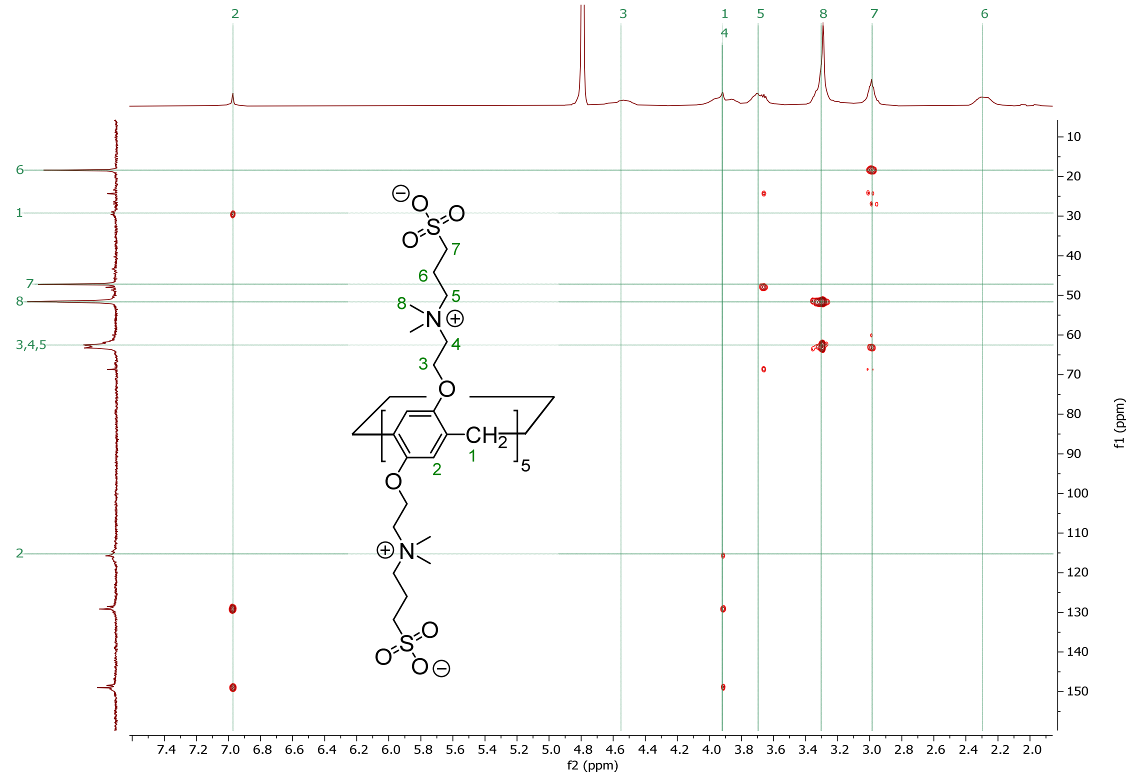

Figure S-12. HMBC (D_2O, 500;126 MHz, 25.0 ºC) obtained for compound **ZP5A**.

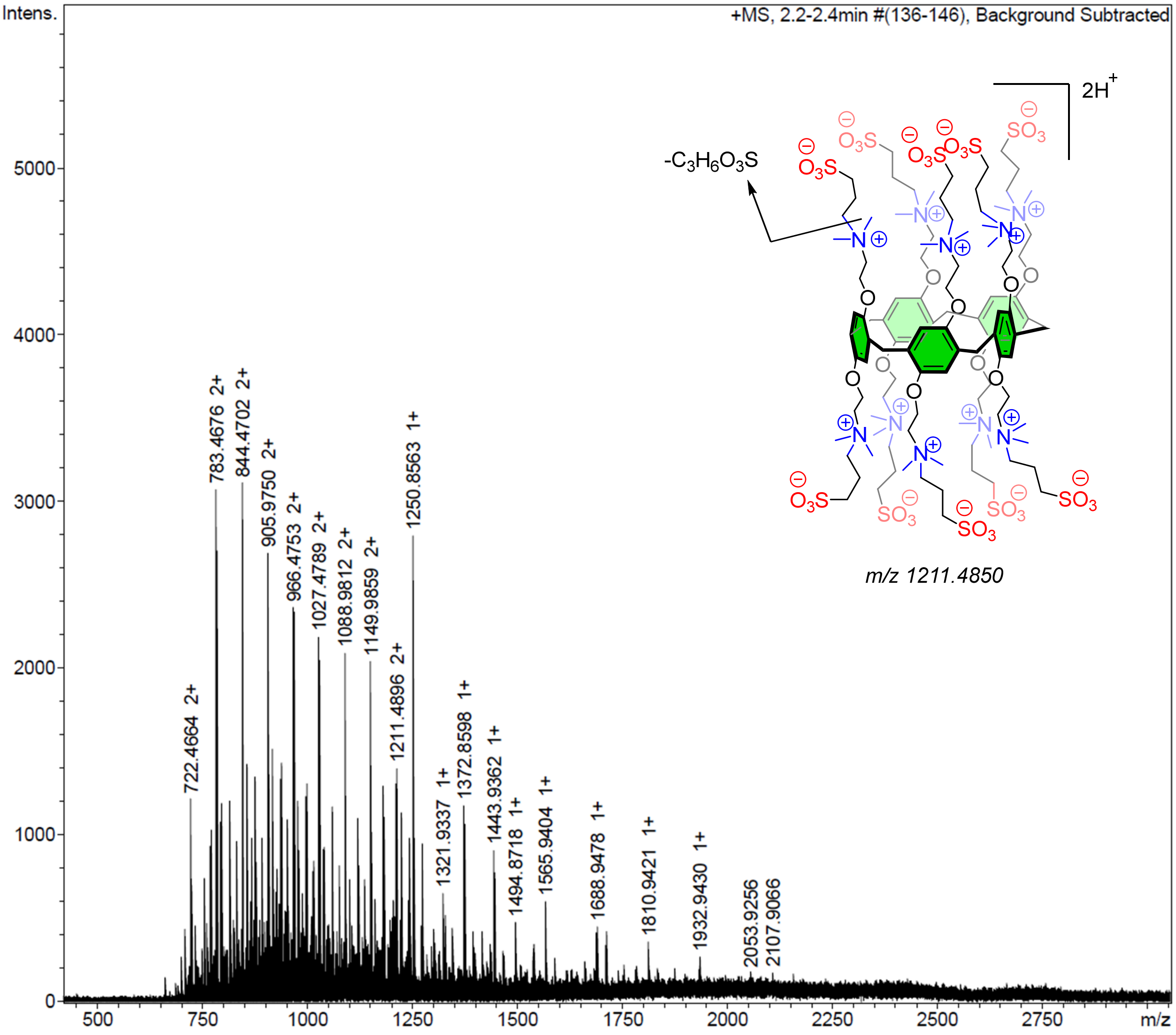

Figure S-13. ESI-MS spectrum obtained for compound **ZP5A**.

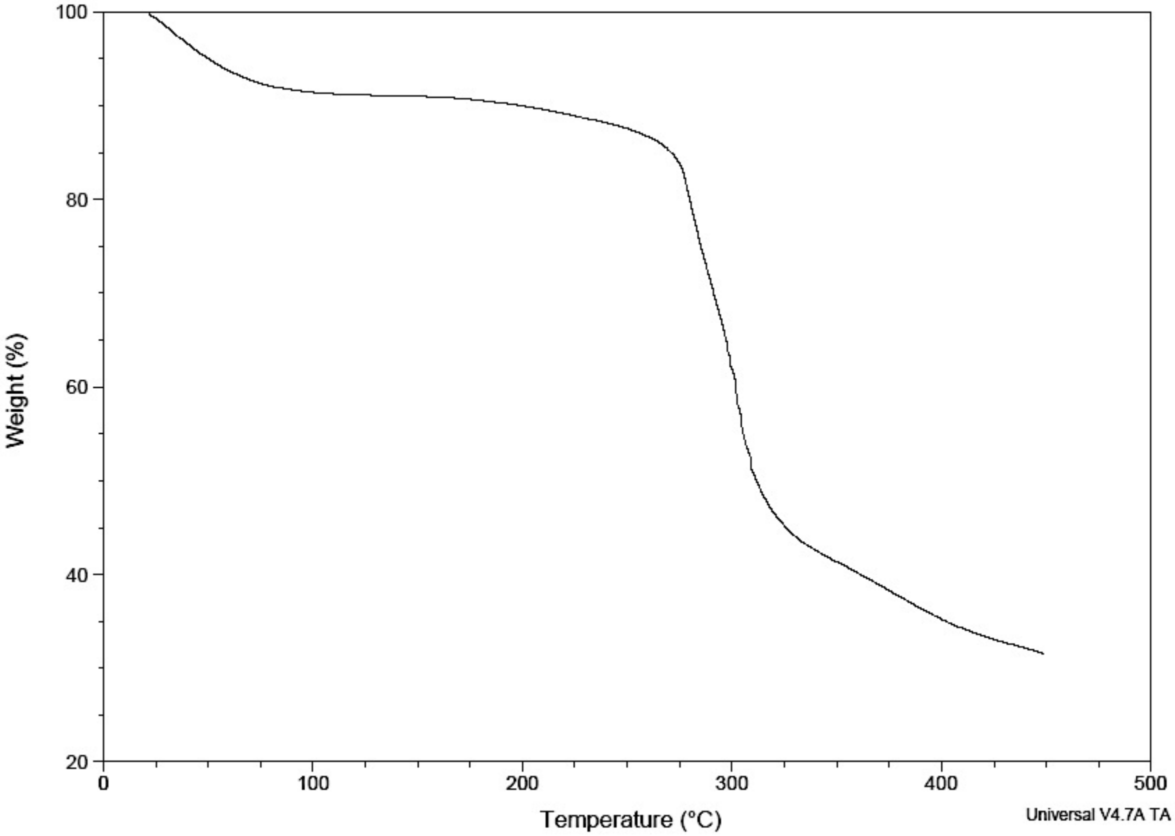

Figure S-14. Thermogravimetric analysis for a sample of **ZP5A** revealing that the zwitterionic pillararene is thermally stable up to 250 °C, being the first weight loss assigned to solvent loss.

3.2. Synthesis of Z3

The synthetic strategy for the preparation of hexyl-(2-(butylammonium)ethyl)phosphate (**Z3**) is outlined in Scheme S-2.

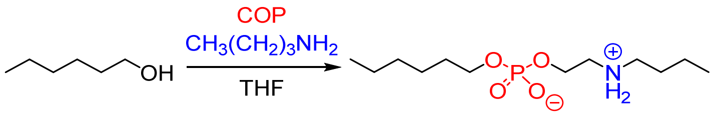

Scheme S-2. Synthetic strategy for the preparation of **Z3**.

The procedure for obtaining **Z3** was carried out using a variant of the method previously described by Kang *et al.*[6] Prior to use, 1-hexanol and *n*-butylamine were dried with Mg/I_2 and calcium hydride, respectively. A solution of 2-chloro-2-oxo-1,3,2-dioxaphospholane (COP) (1.0 g, 6.67 mmol) in 2.0 mL of dry tetrahydrofuran was added dropwise over a cold (0 °C) solution of 1-hexanol (0.84 mL, 6.67 mmol) and diisopropylamine (0.94 mL, 6.67 mmol) in 10 mL of dry tetrahydrofuran under Ar atmosphere and under vigorous stirring. Once the addition was complete, the mixture was stirred for 1 h at 0 °C and for another 2 h at room temperature. Subsequently, *n*-butylamine (2.0 mL, 20 mmol) dissolved in 15 mL of dry tetrahydrofuran was added. Then, the diisopropylamine hydrochloride that precipitated was filtered off, and the filtrate was concentrated. The residue was purified by recrystallization from ethyl acetate/hexane mixtures, providing 0.85 g (45%) of the expected **Z3** as a white solid.

^{1}H NMR ($CDCl_3$, 500 MHz): δ 10.43 (br s, 2H), 4.26 – 4.18 (m, 2H), 3.88 (q, *J* = 6.7 Hz, 2H), 3.16 – 3.11 (m, 1H), 2.87 (t, *J* = 7.9 Hz, 2H), 1.75 (q, *J* = 7.9 Hz, 2H), 1.61 (q, *J* = 7.1 Hz, 2H), 1.40 (h, *J* = 7.4 Hz, 2H), 1.36 – 1.31 (m, 2H), 1.30 – 1.24 (m, 4H), 0.93 (t, *J* = 7.4 Hz, 3H), 0.87 (t, *J* = 6.9 Hz, 3H) ppm.
^{13}C{1H} NMR ($CDCl_3$, 126 MHz): δ 66.6 (CH_2), 60.8 (CH_2), 49.6 (CH_2), 48.1 (CH_2), 32.1 (CH_2), 30.8 (CH_2), 28.1 (CH_2), 25.9 (CH_2), 22.8 (CH_2), 20.1 (CH_2), 14.3 (CH_3), 13.7 (CH_3) ppm.
^{1}H NMR (D_2O, 500 MHz): δ 4.09 (q, *J* = 5.9 Hz, 2H), 3.87 (q, *J* = 6.7 Hz, 2H), 3.30 (t, *J* = 4.7 Hz, 2H), 3.13 – 3.02 (m, 2H), 1.70 – 1.58 (m, 4H), 1.37 (h, *J* = 7.5 Hz, 2H), 1.37 – 1.30 (m, 2H), 1.30 – 1.26 (m, 4H), 0.91 (t, *J* = 7.4 Hz, 3H), 0.85 (t, *J* = 7.0 Hz, 3H) ppm.

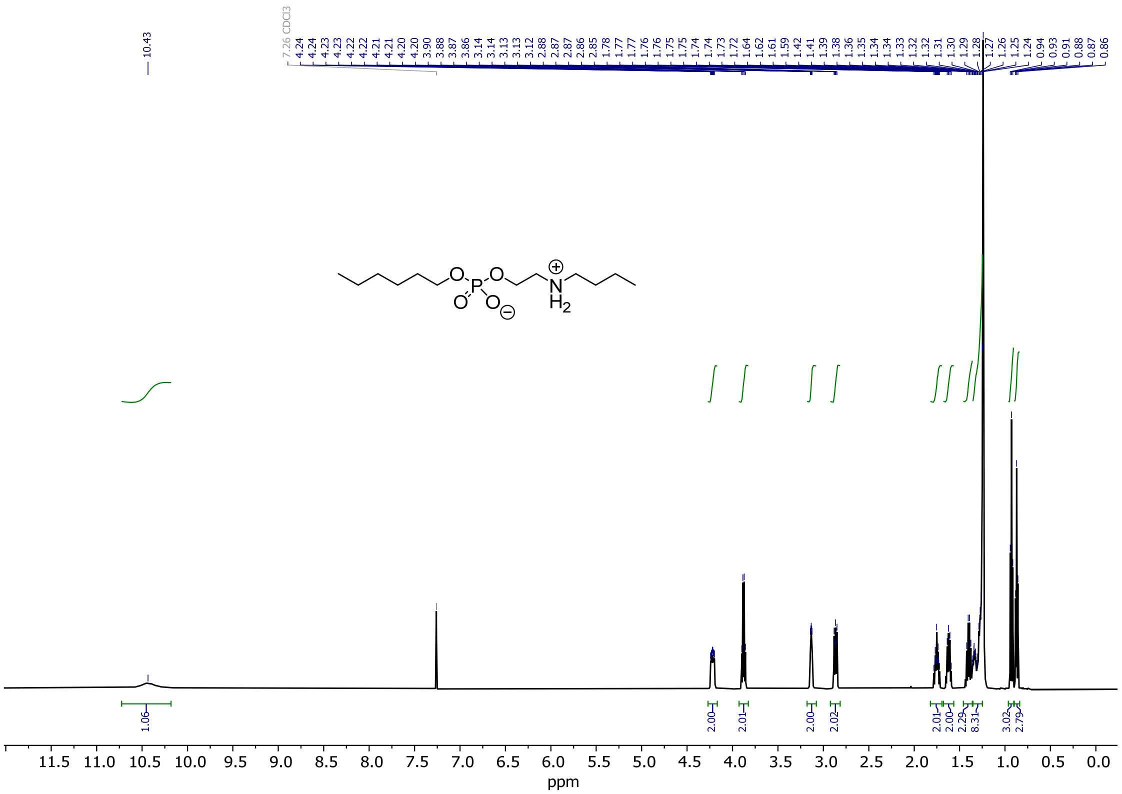

Figure S-15. ^{1}H NMR ($CDCl_3$, 500 MHz, 25.0 ºC) obtained for compound **Z3**.

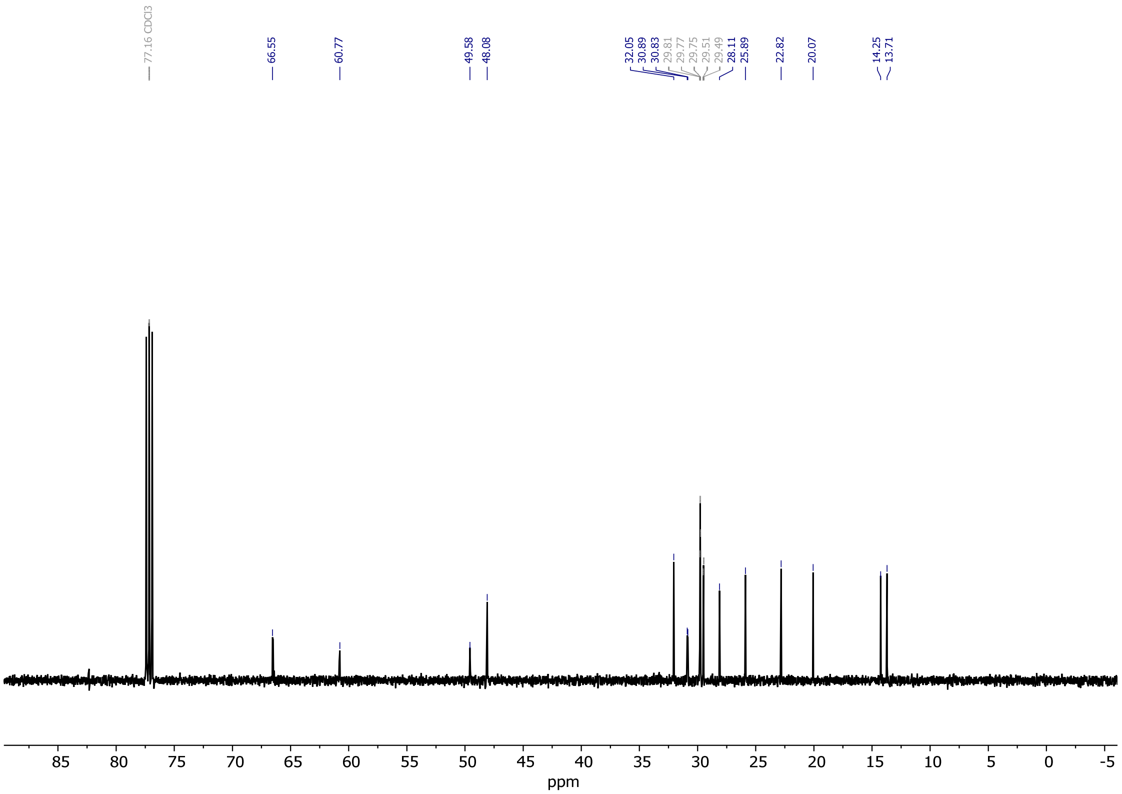

Figure S-16. ^{13}C{1H} NMR ($CDCl_3$, 126 MHz, 25.0 ºC) obtained for compound **Z3**.

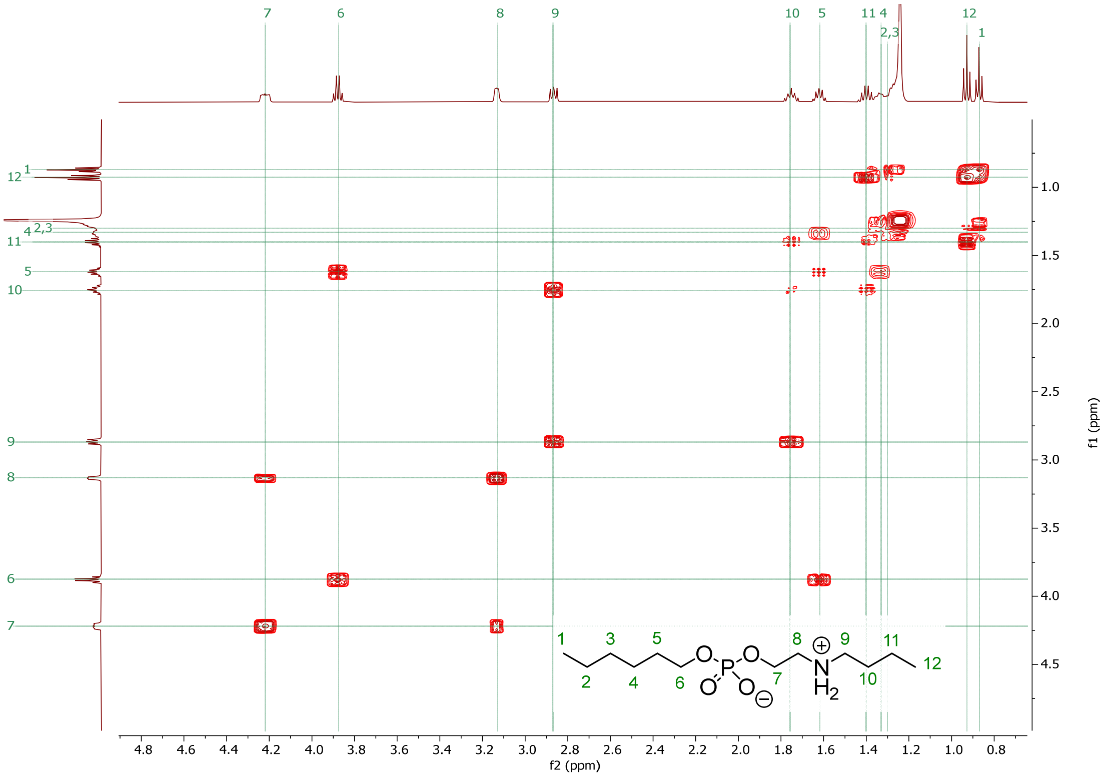

Figure S-17. COSY ($CDCl_3$, 500 MHz, 25.0 ºC) obtained for compound **Z3**.

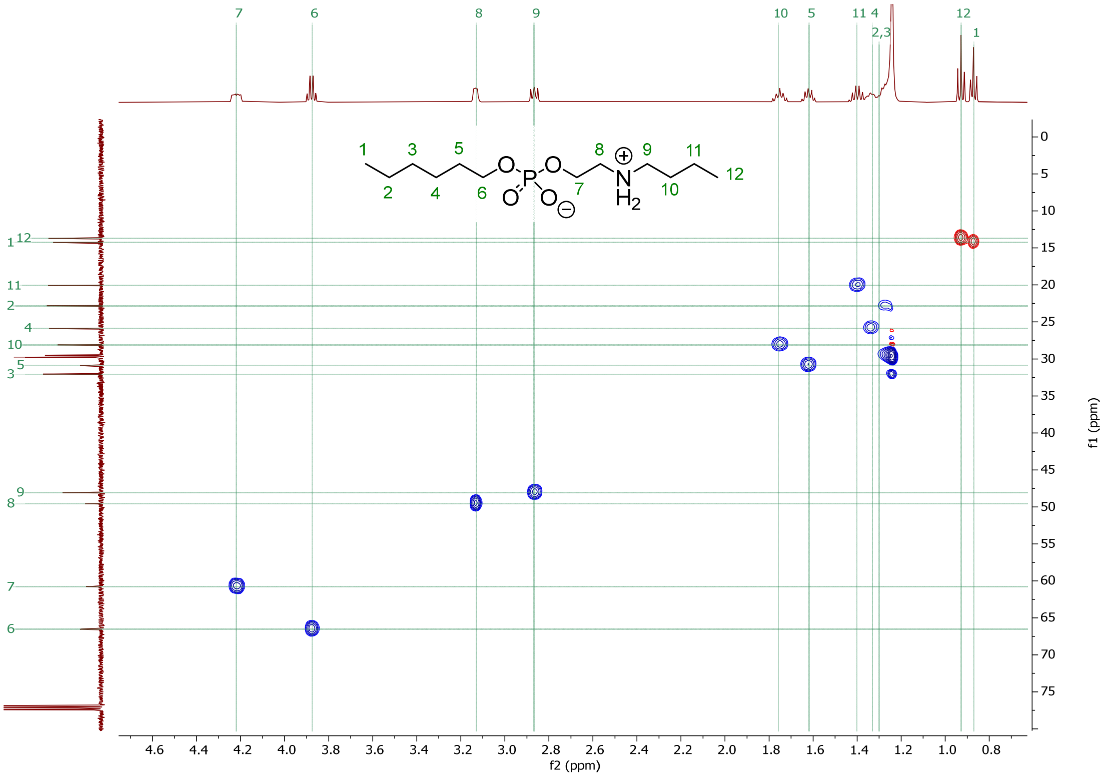

Figure S-18. HSQC ($CDCl_3$, 500;126 MHz, 25.0 ºC) obtained for compound **Z3**.

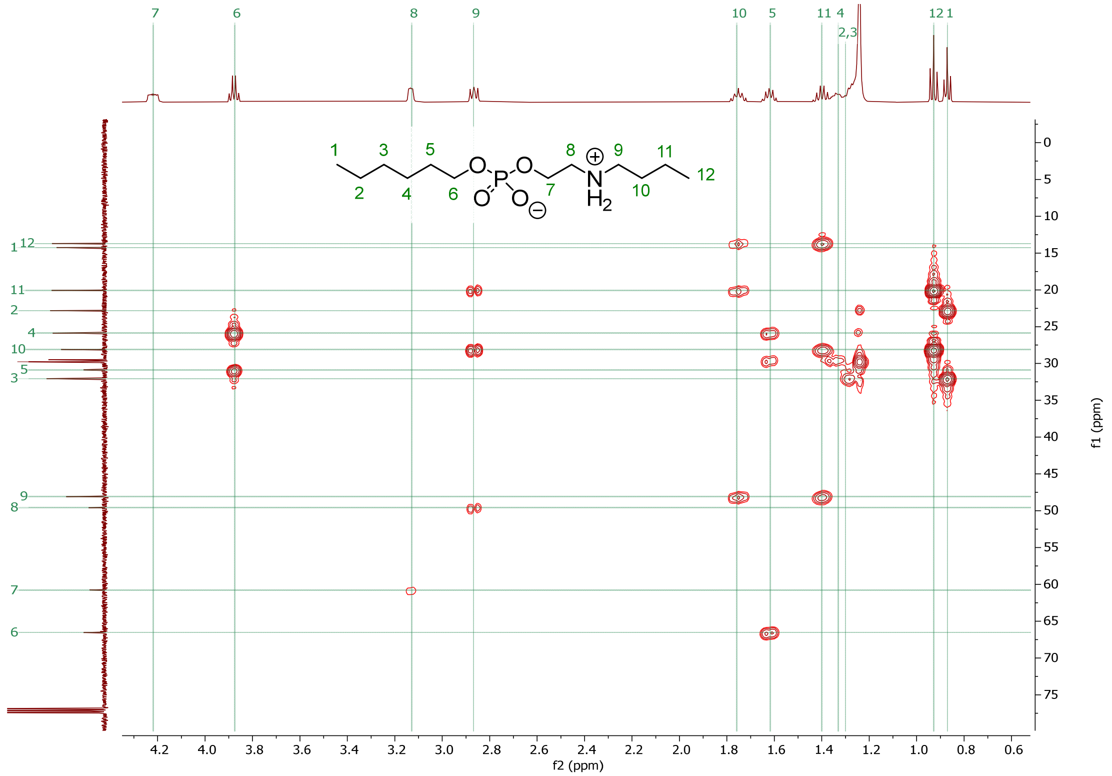

Figure S-19. HMBC ($CDCl_3$, 500;126 MHz, 25.0 ºC) obtained for compound **Z3**.

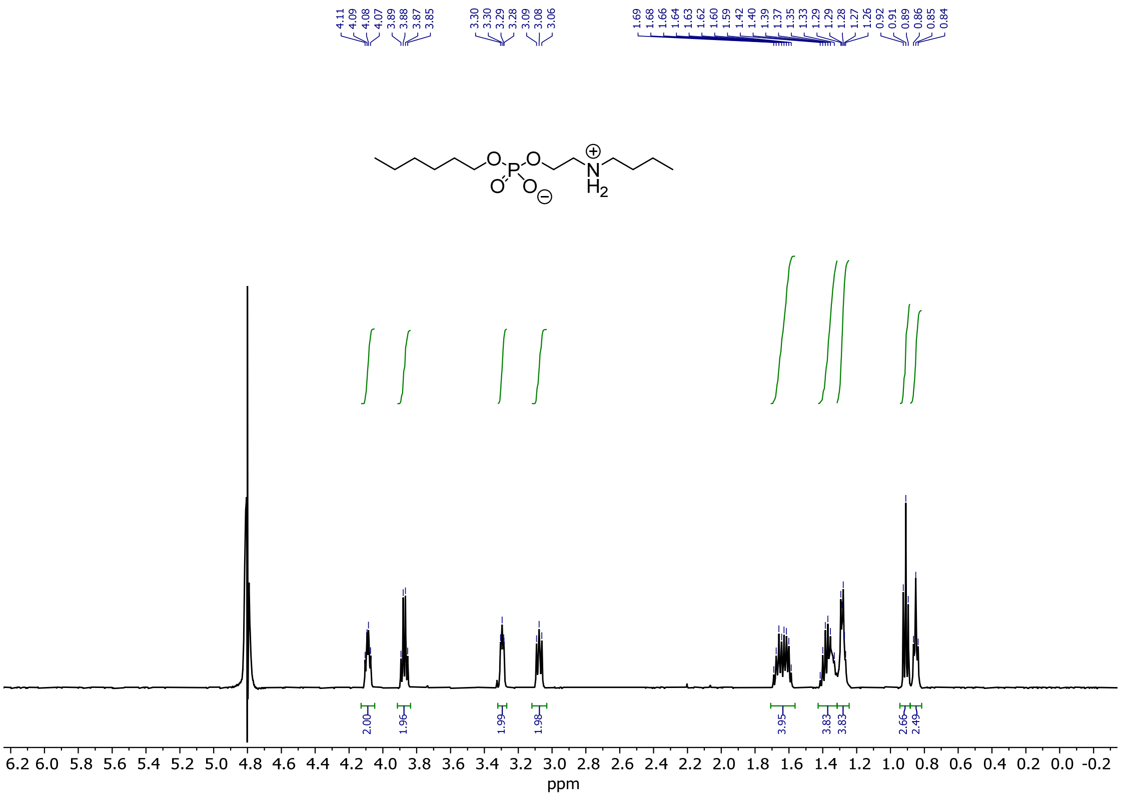

Figure S-20. ^1H NMR (D_2O, 500 MHz, 25.0 ºC) obtained for compound **Z3**.

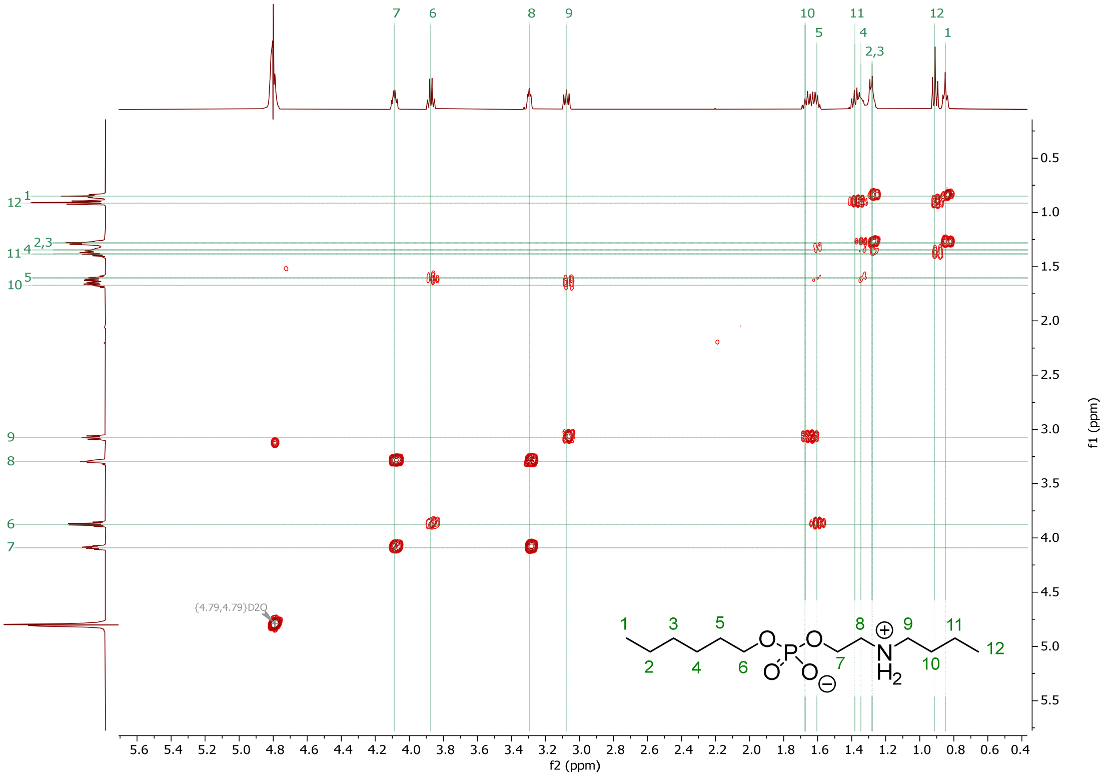

Figure S-21. COSY (D_2O, 500 MHz, 25.0 ºC) obtained for compound **Z3**.

Section 2. Determination of self-diffusion coefficients

To explore the potential for self-aggregation of zwitterionic pillararene (**ZP5A**) due to its molecular structure, DOSY ^{1}H NMR experiments were conducted across a range of **ZP5A** concentrations (Figure S-22 to S-24).

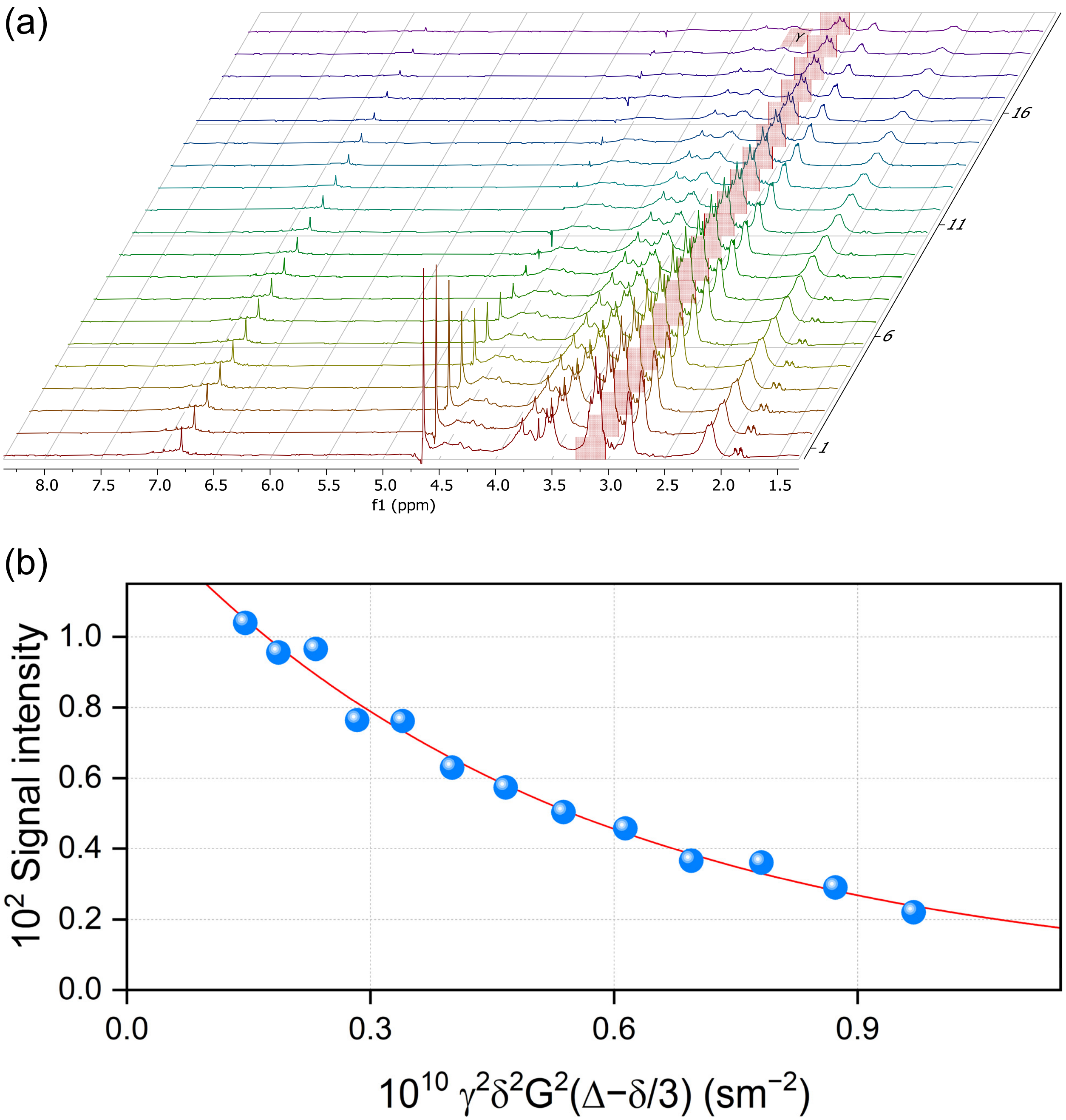

Figure S-22. (a) Signal intensity decay for a series of ^{1}H NMR acquisitions on **ZP5A** in D_2O as a function of gradient strength, and (b) fit to the Stejskal-Tanner equation at δ = 3.11 ppm. [**ZP5A**]=0.5 mM; T=25.0 °C.

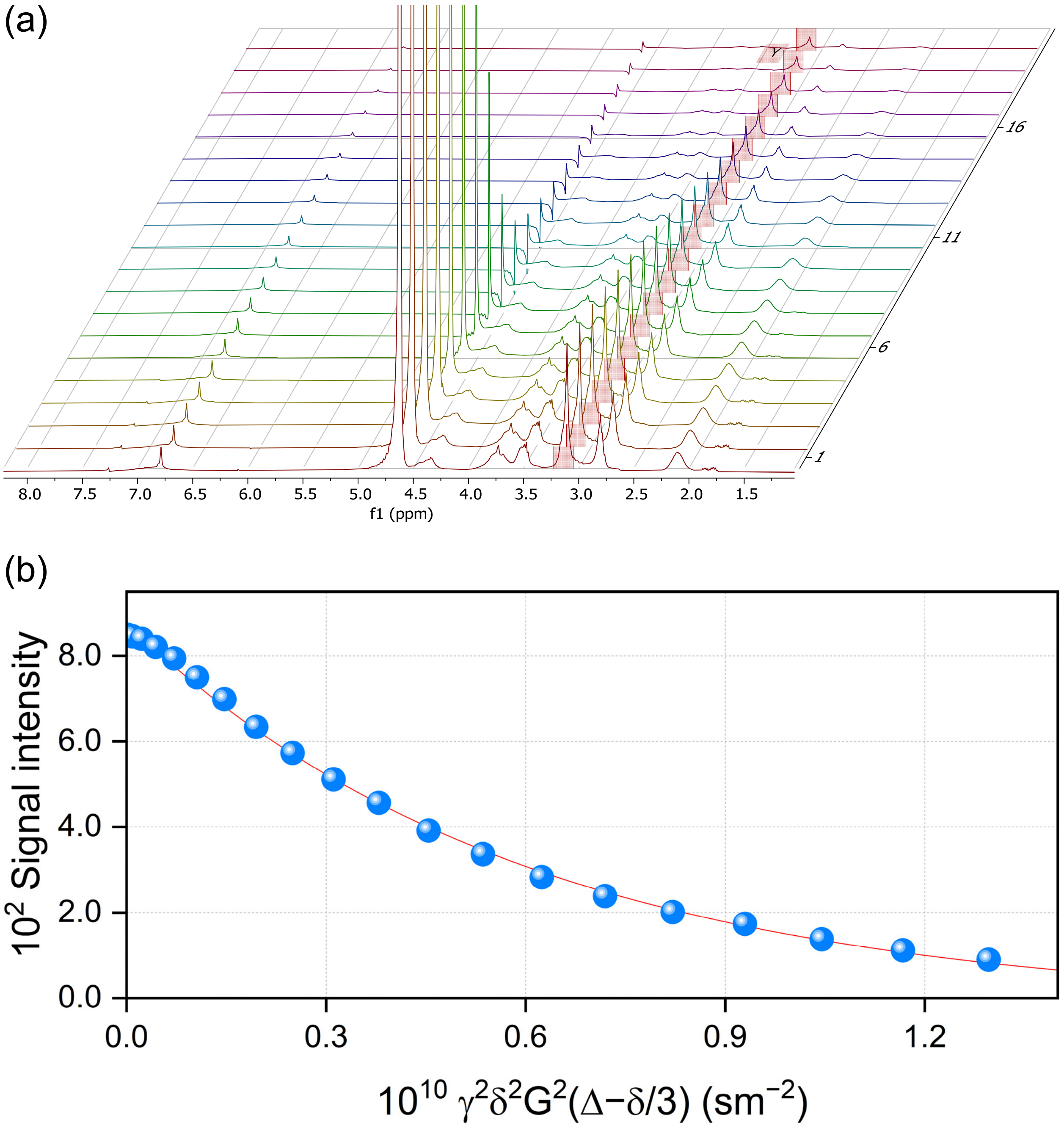

Figure S-23. (a) Signal intensity decay for a series of ^{1}H NMR acquisitions on **ZP5A** in D_2O as a function of gradient strength, and (b) fit to the Stejskal-Tanner equation at δ = 3.11 ppm. [**ZP5A**]=4.3 mM; T=25.0 °C.

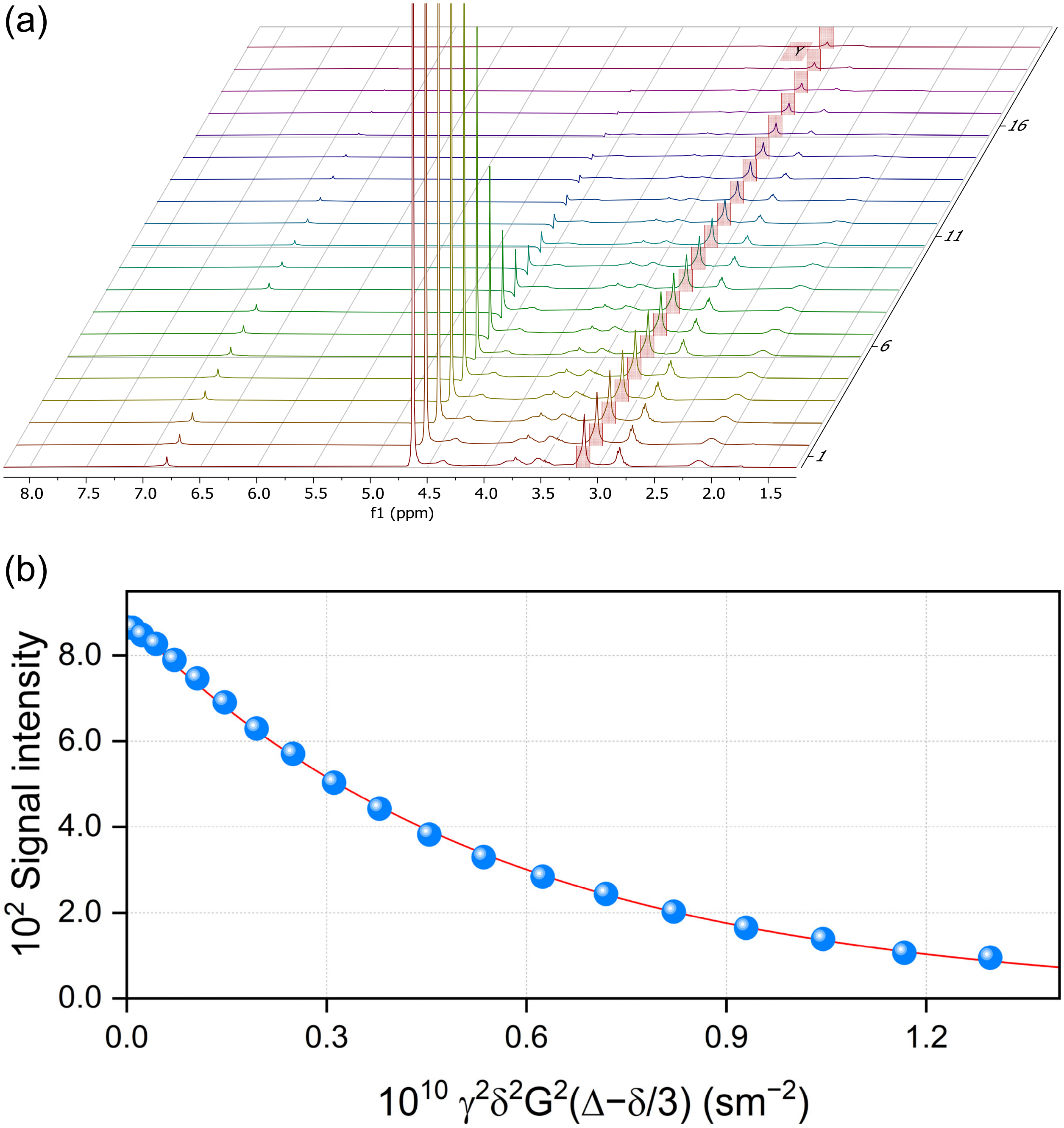

Figure S-24. (a) Signal intensity decay for a series of ^{1}H NMR acquisitions on **ZP5A** in D_2O as a function of gradient strength, and (b) fit to the Stejskal-Tanner equation at δ = 3.11 ppm. [**ZP5A**]=9.8 mM; T=25.0 °C.

As shown in Table S-1, the observed self-diffusion coefficient remains consistent, displaying a slightly lower value than its cationic analogue (**CP5A**) as a result of **ZP5A**'s greater molecular weight. This stable diffusion pattern indicates that **ZP5A** does not undergo self-aggregation under the conditions tested.

Table S-1. Observed self-diffusion coefficients for **ZP5A** and coefficients of **CP5A** (self-diffusion coefficient of water are included as controls). Measurements were taken in D_2O at 25.0 ºC.

**[ZP5A] (M)**	D_{ZP5A} **(m^2s^{-1})**	D_{HDO} **(m^2s^{-1})**	**[CP5A] (M)**	D_{CP5A} **(m^2s^{-1})**[2]
5.0×10^{-4}	$(1.71\pm0.20)\times10^{-10}$	$(1.93\pm0.10)\times10^{-9}$	5.0×10^{-4}	$(2.28\pm0.11)\times10^{-10}$
4.3×10^{-3}	$(1.68\pm0.08)\times10^{-10}$	$(1.96\pm0.10)\times10^{-9}$	1.4×10^{-3}	$(2.28\pm0.11)\times10^{-10}$
9.8×10^{-3}	$(1.81\pm0.09)\times10^{-10}$	$(1.97\pm0.10)\times10^{-9}$	5.2×10^{-3}	$(2.28\pm0.11)\times10^{-10}$

Section 3. Determination of thermodynamic parameters for anionic guests

ITC experiments were conducted to gain insights into the binding mode and thermodynamic parameters, including binding affinity, enthalpy, and entropy, for **ZP5A** and **CP5A** with **TS**⁻ in the absence and presence of external salts (Figure S-25 to S-30).

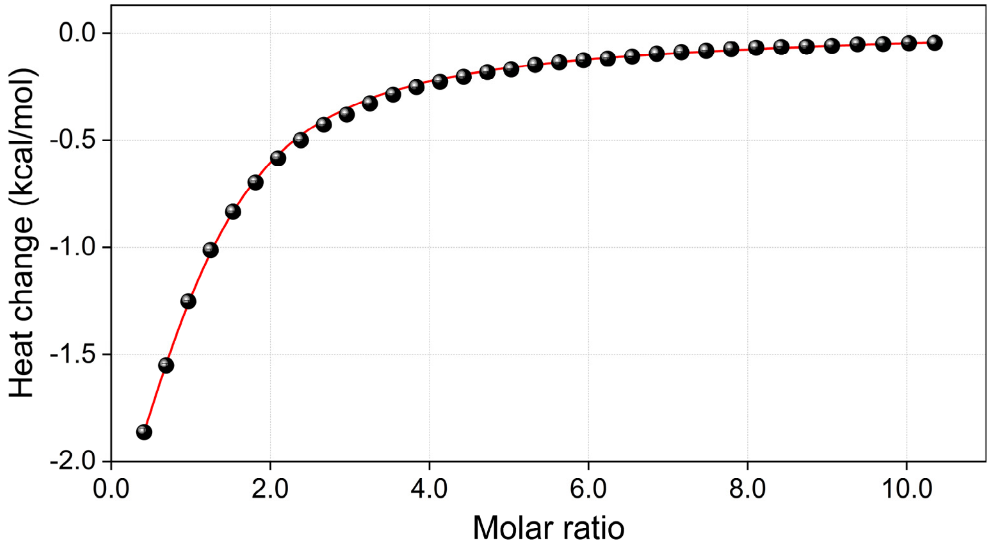

Figure S-25. Integrated heat plot fitted to a 1:1: one-set of binding sites model for the titration of p-toluenesulfonate ([**TS**$^-$]=10.0 mM) into an aqueous solution of pillararene ([**ZP5A**]=0.2 mM) at 25.0 ºC.

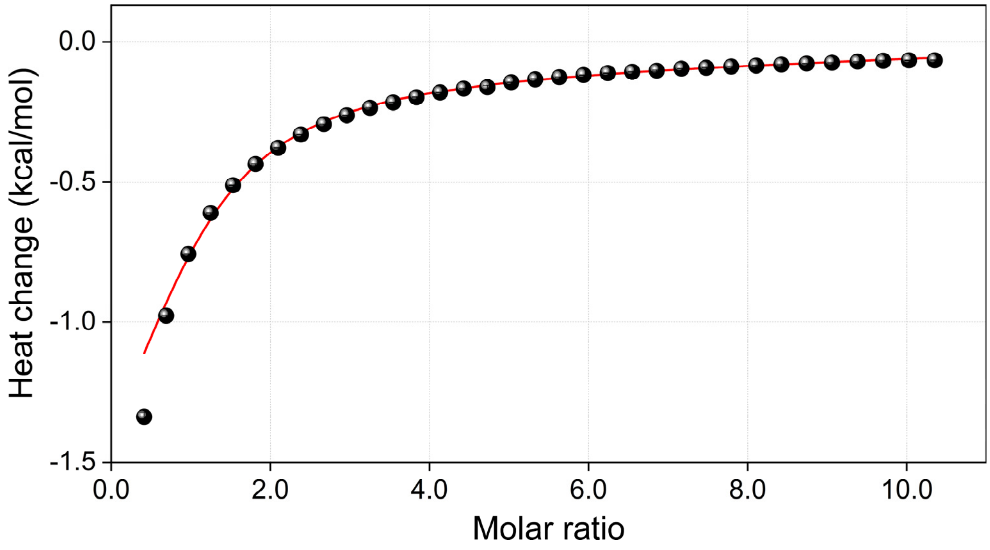

Figure S-26. Integrated heat plot fitted to a 1:1 one-set of binding sites model for the titration of p-toluenesulfonate ([**TS**$^-$]=10.0 mM) into an aqueous solution of pillararene ([**ZP5A**]=0.2 mM) in the presence of sodium bromide ([NaBr]=10.0 mM) at 25.0 ºC.

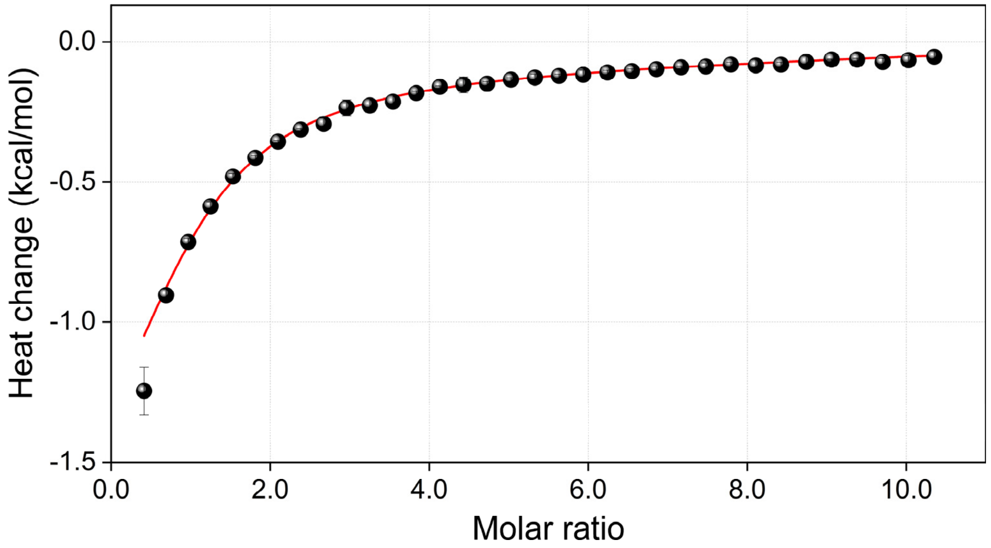

Figure S-27. Integrated heat plot fitted to a one-set of binding sites model for the titration of p-toluenesulfonate ([**TS**$^-$]=10.0 mM) into an aqueous solution of pillararene ([**ZP5A**]=0.2 mM) in the presence of sodium tetrafluoroborate ([$NaBF_4$]=10.0 mM) at 25.0 ºC.

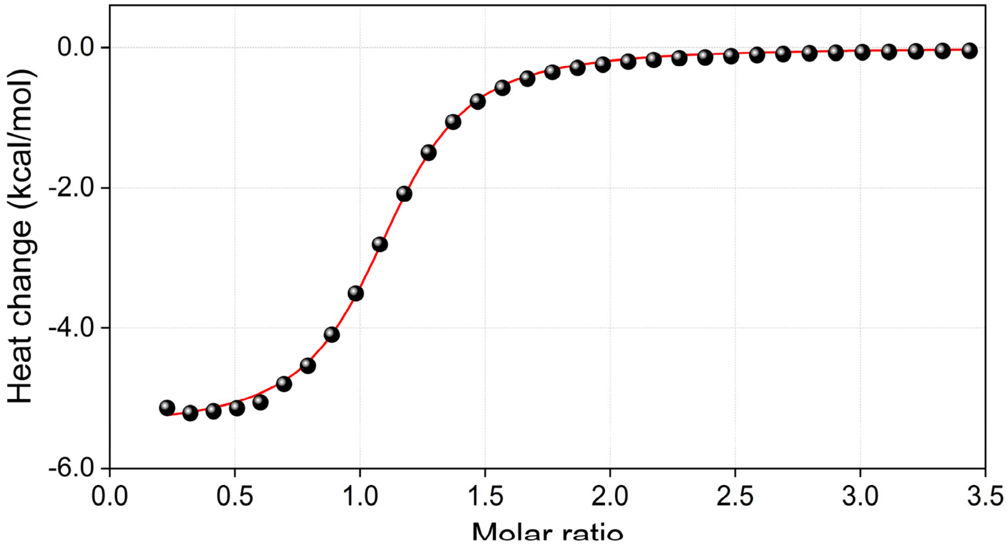

Figure S-28. Integrated heat plot fitted to a one-set of binding sites model for the titration of p-toluenesulfonate ([**TS**$^-$]=10.0 mM) into an aqueous solution of pillararene ([**CP5A**]=0.2 mM, BF_4^- as counterion) at 25.0 ºC.

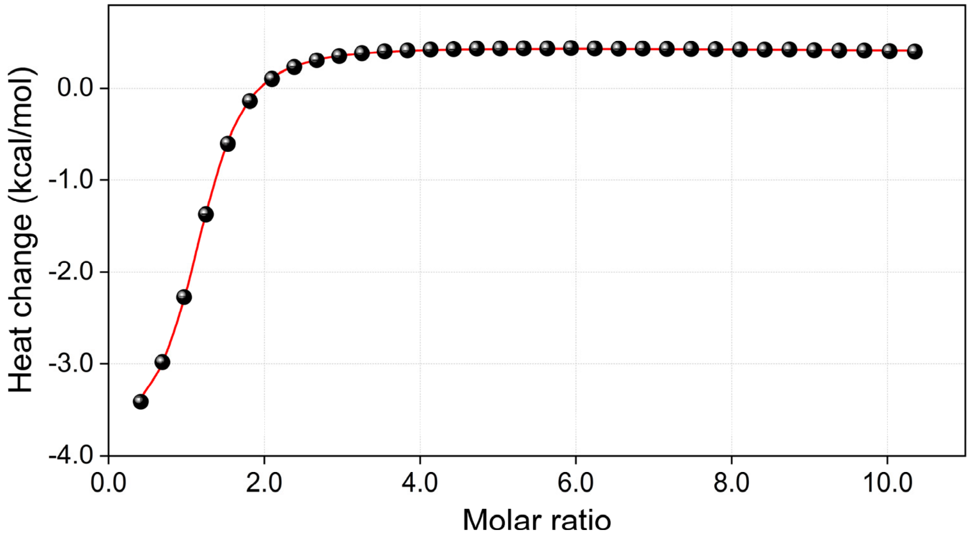

Figure S-29. Integrated heat plot fitted to a one-set of binding sites model for the titration of p-toluenesulfonate ([**TS**$^-$]=10.0 mM) into an aqueous solution of pillararene ([**CP5A**]=0.2 mM, BF_4^- as counterion) in the presence of sodium bromide ([NaBr]=10.0 mM) at 25.0 ºC.

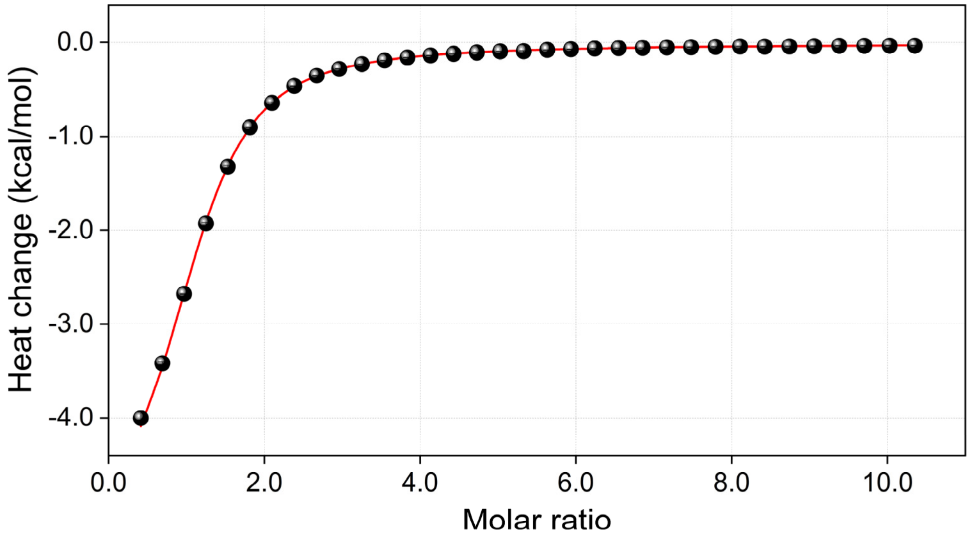

Figure S-30. Integrated heat plot fitted to a 1:1 one-set of binding sites model for the titration of p-toluenesulfonate ([**TS**$^-$]=10.0 mM) into an aqueous solution of pillararene ([**CP5A**]=0.2 mM, BF_4^- as counterion) in the presence of sodium tetrafluoroborate ([$NaBF_4$]=10.0 mM) at 25.0 ºC.

Section 4. Characterization of host-guest interactions

NMR titration experiments were conducted at increasing concentrations of TS^- to determine whether the affinity constant between the zwitterionic pillararene and the anionic guest remains unchanged under biologically relevant conditions ([NaCl]=137 mM). This was assessed by monitoring the chemical shift of the protons located ortho to the sulfonate group of TS^- as the titration progressed (Figure S-31a). As shown in Figure S-31b, the NMR results fitted perfectly to a 1:1 complexation model, yielding a binding constant $K_{ZP5A:TS^-} = (6.7\pm0.8)\times10^3\ M^{-1}$, which is consistent with the value reported in Table 1 in the absence of added salts.

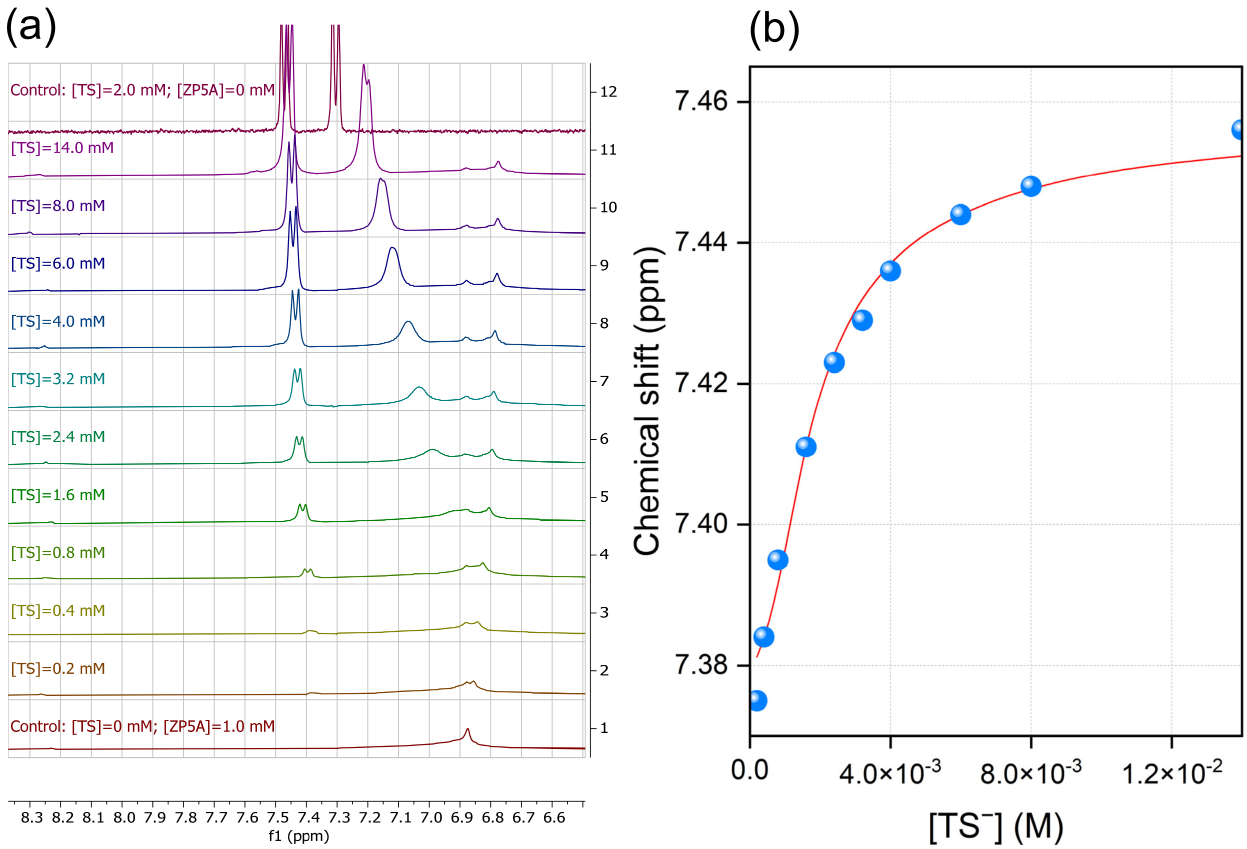

Figure S-31. (a) Set of ^{1}H NMR spectra of solutions containing mixtures of pillar[5]arene (1.0 mM) in the presence of increasing concentrations of *p*-toluenesulfonate at [NaCl]=137 mM in D_2O at 25.0 ºC, and (b) Evolution of the chemical shift of the protons located ortho to the sulfonate group of TS^- as guest concentration is increased, and fit to a 1:1 complexation model (red line).

NMR experiments were conducted to gain insights into the potential molecular interactions, binding sites, and conformational dynamics between **BTA**$^{+}$ and **ZP5A**, revealing no variations in the signals corresponding to the protons of this potential guest (Figure S-32), thereby ruling out its inclusion within the pillararene cavity.

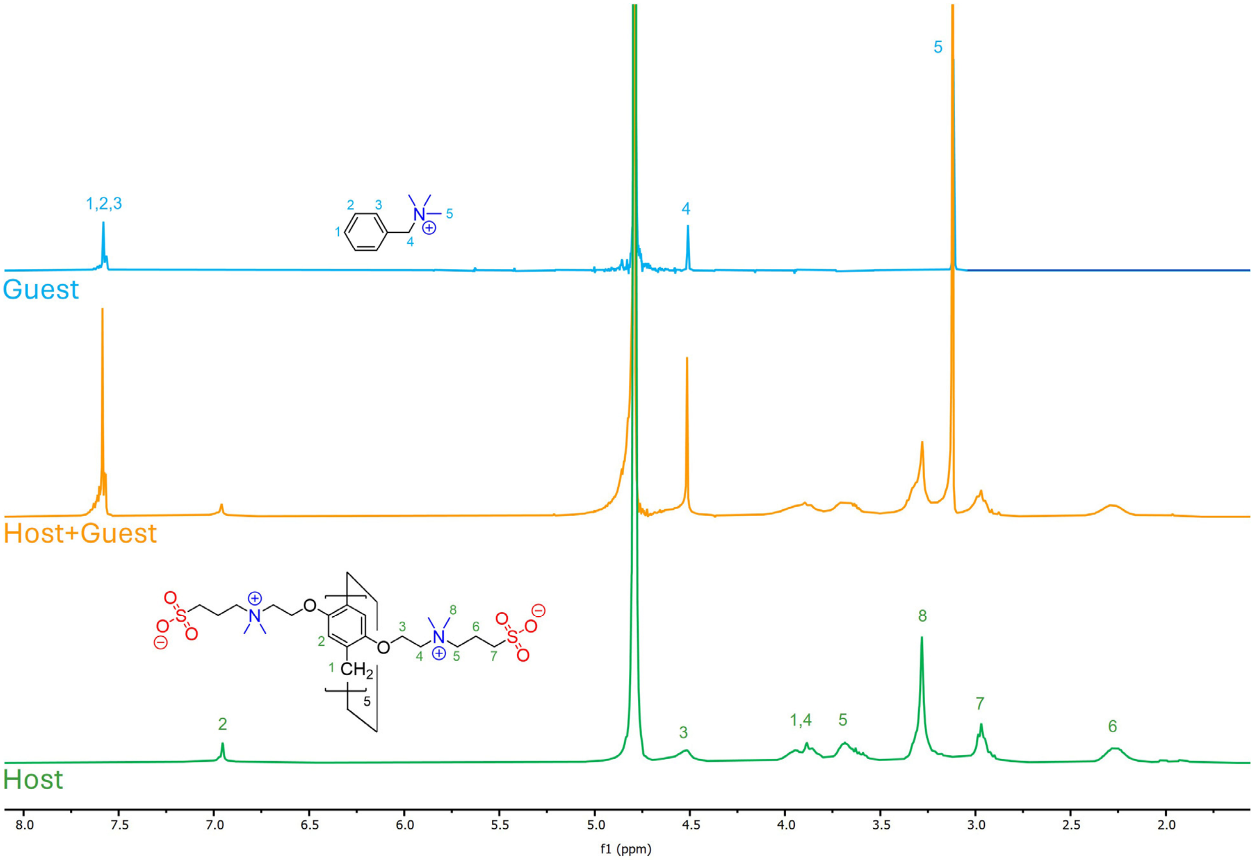

Figure S-32. Set of ^{1}H-NMR spectra in D_2O at 25.0 ºC of: (a) benzyltrimethylammonium ([**BTA**$^{+}$]=4.0 mM), (b) mixture of zwitterionic pillararene ([**ZP5A**]=1.0 mM) and benzyltrimethyl ammonium ([**BTA**$^{+}$]=4.0 mM), and (c) zwitterionic pillararene ([**ZP5A**]=2.0 mM).

Section 5. Determination of thermodynamic parameters for zwitterionic guests

ITC experiments were conducted to gain insights into the binding mode and thermodynamic parameters, including binding affinity, enthalpy, and entropy, for **ZP5A** and **CP5A** with **Z1**, **Z2**, and **Z3** in the absence and presence of external salts (Figure S-33 to S-37).

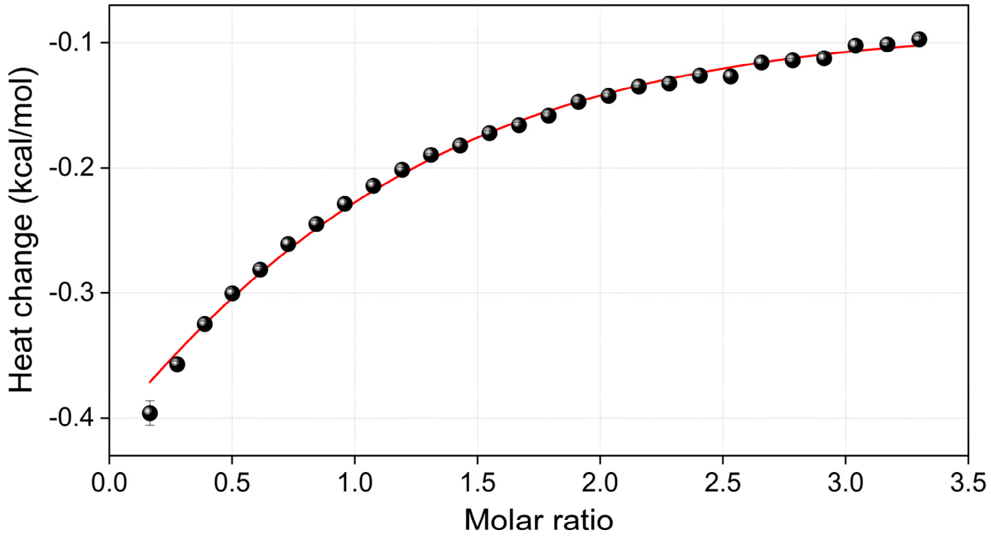

Figure S-33. Integrated heat plot fitted to a 1:1 one-set of binding sites model. for the titration of 3-(N,N-dimethyloctylammonium) propanesulfonate ([**Z1**]=8.0 mM) into an aqueous solution of pillararene ([**ZP5A**]=0.5 mM) at 25.0 ºC.

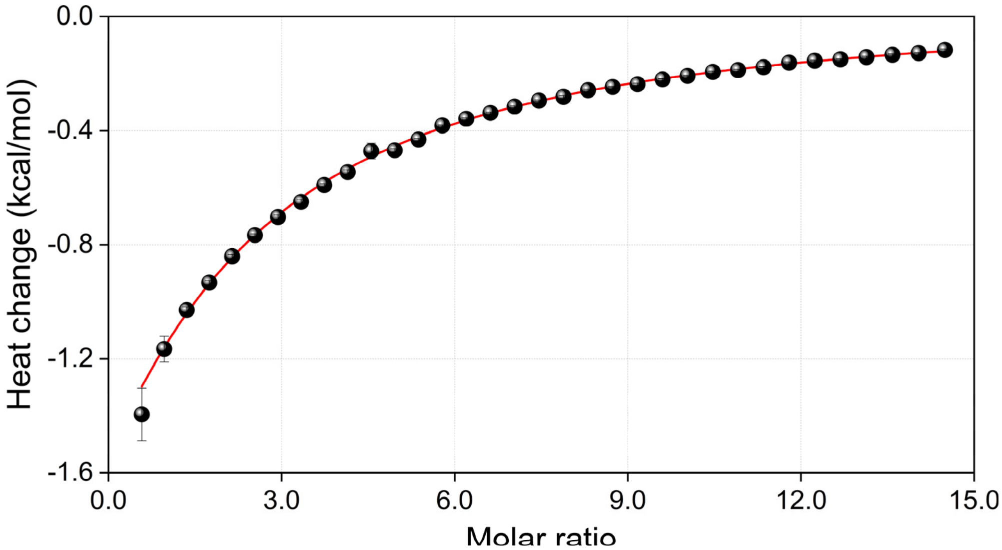

Figure S-34. Integrated heat plot fitted to a 1:1 one-set of binding sites model for the titration of octyl-(2-(trimethylammonium)ethyl) phosphate ([**Z2**]=7.0 mM) into an aqueous solution of pillararene ([**ZP5A**]=0.5 mM) at 25.0 ºC.

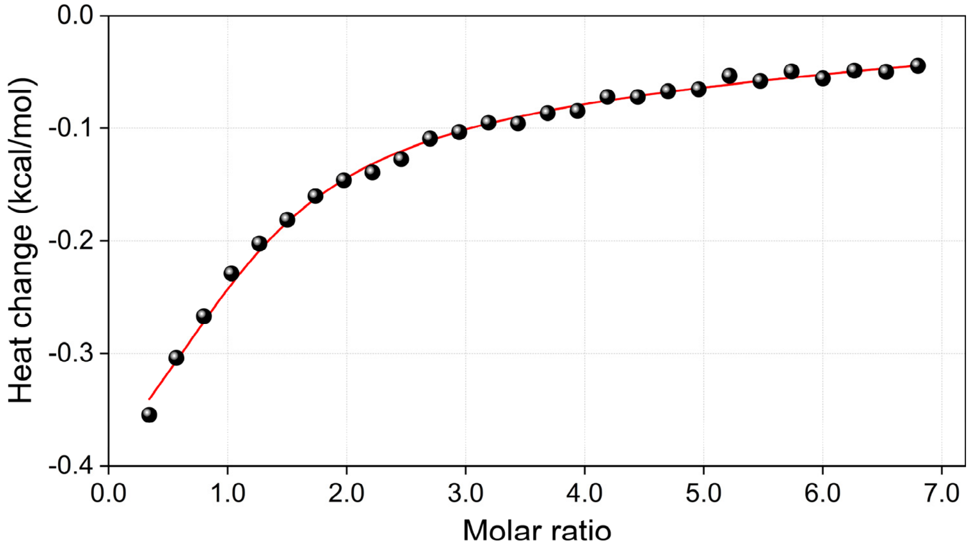

Figure S-35. Integrated heat plot fitted to a 1:1 one-set of binding sites model for the titration of hexyl-(2-(butylammonium)ethyl)phosphate ([**Z3**]=15.0 mM) into an aqueous solution of pillararene ([**ZP5A**]=0.5 mM) at 25.0 ºC.

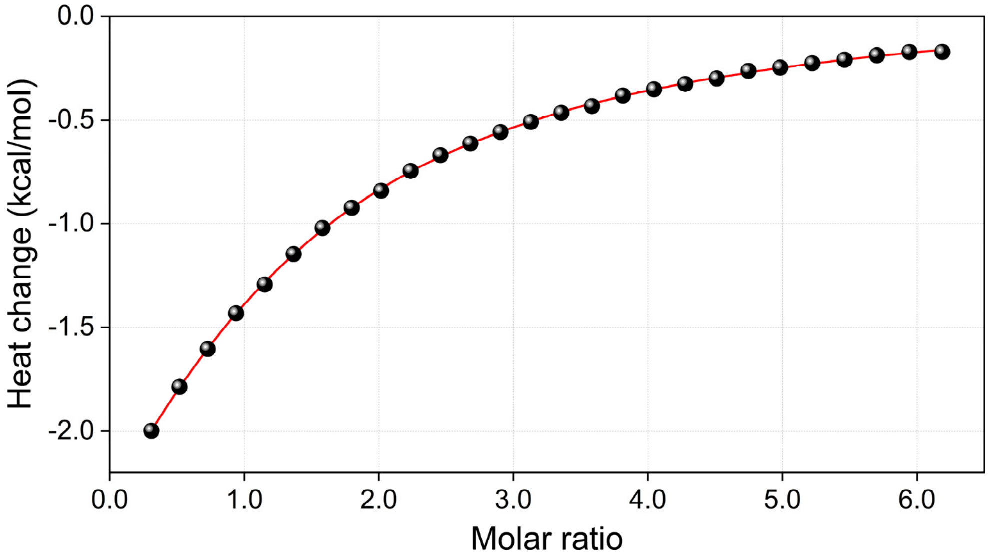

Figure S-36. Integrated heat plot fitted to a 1:1 one-set of binding sites model for the titration of hexyl-(2-(butylammonium)ethyl)phosphate ([**Z3**]=15.0 mM) into an aqueous solution of pillararene ([**CP5A**]=0.5 mM, BF_4^- as counterion) at 25.0 ºC.

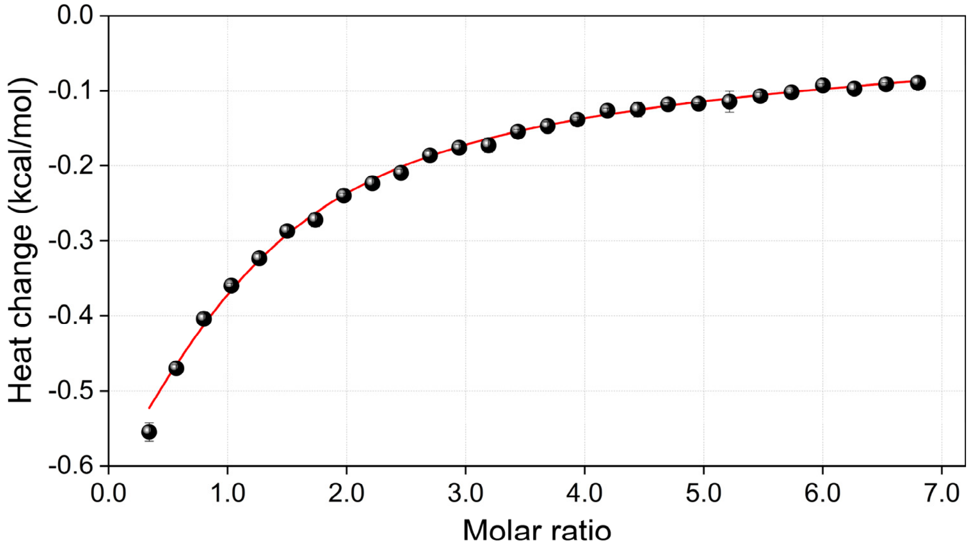

Figure S-37. Integrated heat plot fitted to a 1:1 one-set of binding sites model. for the titration of hexyl-(2-(butylammonium)ethyl)phosphate ([**Z3**]=15.0 mM) into an aqueous solution of pillararene ([**ZP5A**]=0.5 mM) in the presence of sodium tetrafluoroborate ([$NaBF_4$]=50.0 mM) at 25.0 ºC.

Section 6. References

[1] Ma, Y.; Ji, X.; Xiang, F.; Chi, X.; Han, C.; He, J.; Abliz, Z.; Chen, W.; Huang, F. A Cationic Water-Soluble Pillar[5]arene: Synthesis and Host–Guest Complexation with Sodium 1-Octanesulfonate. *Chem. Commun.* **2011**, *47*, 12340–12342.

[2] Gómez, B.; Francisco, V.; Fernández-Nieto, F.; Garcia-Rio, L.; Martín-Pastor, M.; Paleo, M. R.; Sardina, F. J. Host–Guest Chemistry of a Water-Soluble Pillar[5]arene: Evidence for an Ionic-Exchange Recognition Process and Different Complexation Modes. *Chem. Eur. J.* **2014**, *20*, 12123–12132.

[3] Hu, X.-B.; Chen, L.; Si, W.; Yu, Y.; Hou, J.-L. Pillar[5]arene Decaamine: Synthesis, Encapsulation of Very Long Linear Diacids and Formation of Ion Pair-Stopped [2]Rotaxanes. *Chem. Commun.* **2011**, *47*, 4694–4696.

[4] Icke, R. N.; Wisegarver, B. B.; Alles, G. A. β-Phenylethyldimethylamine. *Org. Synth.* **1945**, *25*, 89–91.

[5] Weers, J. G.; Rathman, J. F.; Axe, F. U.; Crichlow, C. A.; Foland, L. D.; Scheuing, D. R.; Wiersema, R. J.; Zielske, A. G. Effect of the Intramolecular Charge Separation Distance on the Solution Properties of Betaines and Sulfobetaines. *Langmuir* **1991**, *7*, 854–867.

[6] Kang, E.-C.; Kataoka, S.; Kato, K. Synthesis and Properties of Alkyl Phosphorylcholine Amphiphiles with a Linear and an Asymmetrically Branched Alkyl Chain. *Bull. Chem. Soc. Jpn.* **2005**, *78*, 1558–1564.
